# Effects of Nixing Pottery Storage on Microbial Community Characteristics, Quality, and Volatile Components of Liupao Tea

**DOI:** 10.3390/microorganisms14092050

**Published:** 2026-09-14

**Authors:** Junru Mao, Yanling Wu, Xianrui Chen, Zhimin Huang

**Affiliations:** Guangxi Key Laboratory of Advanced Microwave Manufacturing Technology, State Key Laboratory of Non-Food Biomass Energy, Guangxi Academy of Sciences, Nanning 530007, China

**Keywords:** Nixing Pottery, food storage, Liupao tea, microbial community, tea quality, volatile components

## Abstract

Liupao tea tended to suffer from unbalanced microbial community structure during storage, which further caused quality deterioration of tea, resulting in economic losses and potential food safety risks. Nixing Pottery (NP) exhibited remarkable advantages in the storage and fresh keeping of tea. Taking glass containers (GC) as the control group, this study investigated the effects of NP on the infusion color, non-volatile internal components, volatile aromatic components, and microbial community structure of Liupao tea during a 24-month storage period. The results indicated that Liupao tea stored in NP showed significant differences compared with that stored in GC. Compared with the tea stored in GC, Liupao tea preserved in NP for 24 months exhibited a reddish-brown appearance with higher brightness, and the degradation rates of non-volatile substances, such as water extracts, free amino acids, and tea polyphenols, were relatively slower. A total of 128 volatile compounds were detected by GC-MS, among which 34 were differential volatile compounds, and these compounds collectively led to the flavor differences between Liupao tea stored in NP and GC. High-throughput sequencing results revealed that Firmicutes and Proteobacteria maintained stable dominant positions for a long time in the NP storage system. Correlation analysis suggested statistical associations between microorganisms, including Firmicutes and Eurotiales, and the transformation of major chemical components in Liupao tea. This study laid a theoretical foundation for the storage and fresh keeping of Liupao tea, the industrial promotion of NP, and the development of new natural mineral-based packaging materials for fermented tea.

## 1. Introduction

Liupao tea is a specialty dark tea from Guangxi, China, and is typically a microbial-fermented tea. Its quality formation mainly relies on microorganisms such as *Eurotium cristatum* and *Aspergillus niger*, which degrade and transform macromolecules, including polyphenols and polysaccharides, thereby producing characteristic flavors and functional components such as theabrownins [1]. As a typical aged tea, Liupao tea relies on prolonged storage to optimize its sensory quality and chemical composition [2]. At present, diverse storage containers are applied in the Liupao tea industry and daily life, yet most conventional storage methods have prominent limitations [3]. In the study of Zhu [4], transparent glass bottles, breathable LDPE films, and open containers were used to investigate the storage effect on tea. The results showed that tea stored in transparent glass bottles presented a reduced oxidation rate, with slight changes in color and tea aroma. For fermented tea, the transparent light environment accelerates the oxidative deterioration of tea internal substances and hinders the normal post-fermentation process of Liupao tea [5]. Plastic bags, tin cans, and aluminum foil bags have excessively high airtightness, which easily causes stuffy and sour tastes of tea and fails to dynamically regulate the internal microenvironment [6,7]. Cartons, wooden boxes, and pottery pots have good air permeability, which is conducive to the aging of fermented tea such as Pu-erh tea. However, the storage effects vary greatly among different containers, and the optimal storage cycle of pottery pots is 12 months [8], so the selection of containers for long-term storage still needs further research. The above drawbacks restrict the quality stability and aging effect of Liupao tea during long-term storage. Xu [9] reported that air permeability, temperature, humidity, and microbial community are key factors affecting the quality evolution of Liupao tea during aging. Therefore, selecting appropriate storage containers and environments is critical to maintaining and improving the overall quality of Liupao tea.

Nixing Pottery (NP) is a national intangible cultural heritage of China. It is produced from unique high-iron red clay and silicon-rich purple-red clay in Qinzhou, Guangxi, and fired under a reducing atmosphere at temperatures above 1200 °C [10]. NP possesses a microporous structure, which endows it with a large specific surface area and the unique property of being air-permeable yet watertight [11]. These characteristics help preserve the nutrients and aroma of tea, making it suitable for storing fermented teas. Besides its structural benefits, the spontaneous polarization property of NP and their related functions have increasingly become a focus of research in recent years. As early as 1989, researchers first discovered the spontaneous polarization effect in natural silicate minerals [12]. Furthermore, Hong [13] confirmed that NP exhibits a surface electric field and high far-infrared emissivity induced by spontaneously polarized silicate minerals. This occurs because silicate precursors in the raw clay undergo a phase transformation during high-temperature firing, ultimately forming the spontaneously polarized crystal KCa_3_AlCa_3_Si_4_O_16_ (hexagonal system, L6 symmetry, P6_3_ space group). This crystal possesses a high spontaneous polarization intensity (0.2322 μC/cm^2^) and far-infrared emissivity (0.951) [14]. Therefore, the presence of spontaneously polarized crystals provides NP with additional favorable functions for tea storage. On the one hand, slight changes in pressure and temperature trigger intense distortion and stretching of the crystals due to spontaneous polarization, exciting certain ions and groups to higher energy levels [15]. When they transfer to lower energy levels, the excess energy is released as far-infrared radiation [16]. This far-infrared radiation can weaken and break hydrogen bonds between water molecules, promoting the depolymerization of large water clusters into smaller ones, and this property may also affect tea [17]. On the other hand, the surface electric field generated by spontaneously polarized crystals can ionize the surrounding air and water, producing more than 5000 negative air ions (NAI) per cm^3^. NAIs at concentrations above 500/cm^3^ are beneficial to human health and possess air-purifying activity, which may positively influence the storage environment of tea [18,19]. Zhang [14] further confirmed the practical use of the spontaneous polarization effect of Nixing Pottery. Adding spontaneously polarized ceramics to water effectively reduces water cluster size and adjusts the water from weakly acidic to neutral within 180 h. Meanwhile, NP has a far-infrared emissivity of 0.951, which can trigger resonance with microorganisms. In tea storage, this property may help regulate molecular motion inside tea leaves and inhibit the growth and reproduction of harmful microorganisms [20]. Additionally, NP is rich in variable-valence metal ions such as Fe^2+^, Fe^3+^, Mn^2+^, and Mg^2+^, which can act as redox mediators to participate in electron transfer reactions [21]. Based on these characteristics, NP can serve as an excellent carrier for tea storage.

As a unique pottery indigenous to Guangxi, NP has a high degree of compatibility with Liupao tea. Current research on NP mainly focuses on its physicochemical properties, structural characterization, and material modification. Although its advantages in food preservation have been well recognized, there is still a lack of systematic studies on the long-term storage of Liupao tea using NP. It remains unclear how the unique structure and spontaneous polarization properties of NP affect the chemical compositions and flavor profiles of tea.

It should be noted that cardboard boxes are the mainstream bulk containers for the industrial-scale warehousing of Liupao tea. However, in humid southern regions, cardboard boxes are seldom used for household storage of loose Liupao tea, where can-type containers are mostly adopted. Meanwhile, under laboratory conditions, cardboard boxes feature unstable air permeability, paper-derived leachates, and background microbial interference, which can readily introduce uncontrollable confounding variables and fail to meet the experimental requirement for a stable control group in this study. Therefore, glass containers (GC) were selected as the control group for structural comparison with NP.

To fill the research gap regarding the effects of NP on the storage-related properties of Liupao tea, this study took Liupao tea as the experimental material, with GC set as the control group. Through a 24-month controlled storage experiment, multidimensional analyses incorporating container material properties, storage microenvironment, microbial communities, non-volatile components, and volatile components were performed to decipher the dynamic changes of Liupao tea during storage. Furthermore, the correlations between core microorganisms and tea quality components were analysed. This study clarifies the advantages and action mechanisms of NP in Liupao tea storage and provides practical technical references for the scientific storage and quality maintenance of Liupao tea. It also offers new theoretical support for the innovative application and industrial development of Nixing Pottery, a national-level traditional intangible cultural heritage. In addition, this work may inspire new ideas for developing packaging materials derived from natural minerals.

## 2. Materials and Methods

### 2.1. Tea Samples and Chemicals

Liupao tea samples were collected from a standardized commercial tea factory in Cangwu County, Wuzhou City, Guangxi, China, the core producing area of geographical-indication Liupao tea. All raw tea materials were harvested in spring 2023 and processed strictly in accordance with the official local standard DBS45/057-2018, adopting unified post-fermentation, pile fermentation, and drying procedures. To eliminate batch-to-batch variation in raw tea quality, microbial background, and processing differences, all tea materials were fully homogenized and mixed into a single unified batch before the storage experiment. This unified batch design ensured high experimental consistency and enabled precise comparison of storage container effects (NP vs. GC) by excluding raw material heterogeneity. Prior to the experiment, the homogenized tea samples were stored at −20 °C. Low-temperature freezing effectively inhibited enzymatic browning, microbial proliferation, and lipid oxidation; minimized color deterioration; and maintained the initial physicochemical and microbial characteristics of Liupao tea before storage treatment. Three biological replicates were set for each treatment at each sampling time point.

Methanol and acetonitrile (chromatographic grade) were purchased from Sangon Biotech (Shanghai) Co., Ltd. (Shanghai, China). Sodium hydroxide, hydrochloric acid, concentrated sulfuric acid, potassium dihydrogen phosphate, sodium dihydrogen phosphate, lead subacetate, sodium carbonate, ascorbic acid, glucose, gallic acid, and ethanol were obtained from Sinopharm Chemical Reagent Co., Ltd. (Shanghai, China). L-theanine (>98%), caffeine (>98%), catechins (>98%), Folin-phenol, EDTA-2Na, phenol, and ninhydrin hydrate (all analytical grade), as well as aerobic plate count test strips (HP001, Handy Plate) purchased from Guangxi Junnuo Biotechnology Co., Ltd. (Nanning, China).

### 2.2. Experimental Operation

The same batch of Liupao tea samples was fully mixed. The tea leaves were evenly loaded into cleaned and sterilized Nixing Pottery containers (NP) and glass containers (GC), with 4.50 kg of tea leaves filled into each single container (provided by Guangxi Qinzhou Kangyisheng Nixing Pottery Manufacturing Co., Ltd. (Qinzhou, China); NP: height 28 cm, inner diameter 22 cm, volume approximately 10.5 L; GC: height 35 cm, inner diameter 19.5 cm, volume approximately 10.5 L). Before screwing the lid for sealing, the mouth of each container was uniformly sealed with clean and sterile white canvas. All containers were placed in a dry storage room at room temperature, 50 cm above the ground, with a spacing of 10 cm between adjacent containers. All containers were placed in a clean, empty room for storage. Throughout the whole 24-month storage period, temperature and relative humidity were monitored and recorded once every 3 months using a calibrated thermo-hygrometer.

A destructive-sampling experimental design with independent containers was adopted in this study. At each sampling time point, three biologically independent replicate containers were prepared for each storage treatment (NP and GC), resulting in six dedicated storage containers per sampling event, with a total of 30 containers throughout the trial. Fresh independent containers were sampled at different storage time points, and repeated sampling from the same container was not performed during the whole experiment. Sampling was conducted at five time points: 0-month storage (initial control group, CK), 6 months, 12 months, 18 months, and 24 months. Approximately 500 g of tea sample was collected from each storage container while wearing disposable sterile gloves. Each sample was divided into two subsamples and immediately frozen at −20 °C. One subsample was used for the determination of physicochemical indicators of tea, and the other was used for microbial sequencing analysis.

Water storage test: 9 L of deionized water was poured into each NP and GC container, respectively, and three biological replicates were set for each group. The water storage containers were basically consistent with the tea storage containers in appearance and volume, while a water outlet was arranged at the bottom of each water storage container, and sampling could be performed by turning on the valve without opening the bottle cap. Water samples were collected sequentially at seven time points: initial storage stage (CK, 0 d), 10 min, 1 h, 12 h, 24 h, 3 d, and 7 d. A total of 10 mL of water sample was collected each time and transferred into a 50 mL centrifuge tube. All collected water samples were immediately frozen at −20 °C for subsequent physicochemical determination.

### 2.3. Determination of Color Difference in Tea Samples

Tea leaves were ground uniformly into fine powder with a laboratory grinder and sieved through a 40-mesh sieve to guarantee homogeneity. A 3.00 g sample was placed into a beaker, and 150 mL of boiling water was added. After 5 min of infusion, the tea liquor was poured into a container. The L*, a*, and b* values of the tea liquor were measured using a colorimeter (CR-10 Plus, Konica Minolta Co., Ltd., Tokyo, Japan). The L* value represented lightness (a higher value indicated greater brightness); the a* value reflected the color change from green (+) to red (−); the b* value indicated the color change from blue (−) to yellow (+) [22].

### 2.4. Determination of Non-Volatile Components in Tea Samples

The content of water extracts (WE) was determined according to GB/T 8305-2013 (tea determination of water extracts content). Total free amino acids (FAA) were measured using the ninhydrin colorimetric method as described in GB/T 8314-2013 (tea determination of free amino acids content). The contents of tea polyphenols (TP) and catechins (CAT) were analyzed in accordance with GB/T 8313-2018 (determination of total polyphenols and catechins content in tea). Caffeine (CAF) content was determined following GB/T 8312-2013 (tea determination of caffeine content). Tea polysaccharides (TPS) were quantified by the phenol-sulfuric acid method [23].

### 2.5. Determination of Colony Count and Full Width at Half Maximum

Water sample colony counting method: A pipette with sterile tips was used to aspirate 1 mL of water sample, which was spread evenly onto total-bacterial-count test strips. When necessary, the sample was serially diluted by 10- to 100-fold. The test strips were subsequently incubated at 27 °C for 48 h. The full width at half maximum (FWHM) of ^17^O-NMR was tested based on the method described in T/BJWA 005-22. A total of 9.00 L of deionized water was transferred into NP and GC containers, respectively. Water samples were subjected to ^17^O-NMR characterization, and FWHM values were determined at 0 (initial control group, CK), 10 min, 60 min, 12 h, and 24 h. All NMR spectra were acquired on a nuclear magnetic resonance spectrometer (Bruker AVANCE NEO III 600 MHz, Sweden) at room temperature, with deuterated chloroform used as the internal standard [24].

### 2.6. Determination of Volatile Compounds in Tea Samples

A 1.0000 g portion of tea powder was weighed and placed into a 15 mL headspace extraction vial. Then 0.7 g NaCl was added, and the vial was sealed immediately. The vial was placed on a 60 °C thermostatically controlled magnetic stirrer (MS-H-ProT, DLAB, China) for 20 min of equilibration. A fiber extraction head (50/30 µm DVB/CAR/PDMS, Superco, USA), which had been preheated at 250 °C for 20 min, was inserted into the vial for headspace adsorption for 30 min. Afterward, the extraction head was inserted into the injection port of a gas chromatography-time-of-flight mass spectrometer (7890B, Agilent, USA) and desorbed at 250 °C for 5 min to separate and detect volatile components [25].

Instrument parameters: Column: DB-5 MS 30 m × 0.25 mm × 0.25 µm (Agilent, USA); GC conditions: Inlet temperature 250 °C, GC-MS interface temperature 250 °C, carrier gas flow rate 1.0 mL/min; Temperature program: initial 40 °C, hold for 5 min, ramp 5 °C/min to 250 °C, and hold for 10 min. MS conditions: Ion source temperature 230 °C, transfer line temperature 250 °C, injection current 35 µA, EI ionization at 70 eV, full scan from 35-550 da. Retention index (RI) determination: Employed C7-C40 mixed standard detection. Diluted a 1000 µg/mL n-alkane mixed standard solution to 10 µg/mL using n-hexane (chromatography grade). Injected 1 µL of the standard solution at 10 µg/mL into the GC-MS. Calculated the retention index of volatile components using the average value.

A C7-C40 n-alkane mixed standard solution was diluted to 10 µg/mL with chromatographic-grade n-hexane, and 1 µL of the diluted solution was injected for retention index (RI) calibration. Compound identification was performed by matching acquired mass spectra with the NIST database using a spectral match threshold of 75. An RI deviation within ±20 Kovats units was considered acceptable. Compounds eluting before n-heptane (RI < 700) were identified based on mass spectral features and published fragment information due to unavailable RI calibration. Notably, no internal standard or external standard calibration curve was applied in this study. All volatile compound data were calculated by relative peak area normalization and represent relative peak area percentages rather than absolute concentrations. Therefore, data analysis in this study was defined as relative peak area comparative analysis. Owing to differences in ionization efficiency among individual volatile compounds, direct quantitative comparison across different compounds is invalid. All comparative analyses in this study were strictly limited to relative changes of the same compound among different storage groups and time points to evaluate compound accumulation or loss.

### 2.7. SEM Structural Characterization of Tea Samples

A field emission scanning electron microscope (SEM, Thermo Scientific Apreo 2c, Thermo Fisher Scientific, Waltham, MA, USA) was used to observe the microstructure of samples. An Ultim Max 65 energy-dispersive spectrometer (EDS) was adopted to analyze elemental composition. The crystal structure was characterized by X-ray diffraction (XRD, Rigaku D/MAX 2500V, Japan) with Cu Ka radiation (0.154 nm) at 40 kV and 30 mA and a scanning rate of 10° min^−1^. Fourier transform infrared (FTIR) spectra were collected using an FTIR-8400S spectrometer (Shimadzu, Japan) at a resolution of 4 cm^−1^ [14].

### 2.8. DNA Extraction and Sequencing

Total genomic DNA of Liupao tea samples was extracted with the TGuide S96 Magnetic Bead-Based Soil/Fecal Genomic DNA Extraction Kit (DP812, TIANGEN Biotech (Beijing) Co., Ltd. (Beijing, China)), and the extracted DNA was stored at −20 °C for subsequent use. The concentration of DNA was quantified using a Synergy HTX microplate reader with 1× dsDNA HS Working Solution (Yeasen Biotechnology (Shanghai) Co., Ltd. (Shanghai, China)). Qualified DNA templates were subjected to full-length PCR amplification of bacterial 16S rRNA gene and fungal ITS gene, respectively. For full-length 16S rRNA gene amplification, the forward primer 27F (5′-AGRGTTTGATYNTGGCTCAG-3′) and reverse primer 1492R (5′-TASGGHTACCTTGTTASGACTT-3′) were adopted, and a 20 μL reaction system was prepared with KOD One™ PCR Master Mix (KMM-101, Beijing Bio-Boat Biotechnology Co., Ltd. (Beijing, China)). For full-length ITS gene amplification, the forward primer ITSF (5′-CTTGGTCATTTAGAGGAAGTAA-3′) and reverse primer ITSR (5′-TCCTCCGCTTATTGATATGC-3′) were used, and a 15 μL reaction system was prepared with KOD FX Neo (TOYOBO) (KFX-201S, Beijing Bio-Boat Biotechnology Co., Ltd. (Beijing, China)). PCR amplicons were screened via agarose gel electrophoresis to retain qualified samples, followed by purification using Agencourt^®^ AMPure^®^ XP magnetic beads (A63882, Beckman Coulter Life Sciences). All purified samples were preserved at −20 °C and delivered to Tsingke Biotechnology Co., Ltd. (Shanghai, China) for full-length third-generation high-throughput sequencing on the PacBio Sequel II platform.

### 2.9. Bioinformatics Analysis Parameters

Sequencing was performed on the PacBio Sequel II platform. Raw subreads generated from sequencing were corrected using SMRT Link v8.0 software to produce circular consensus sequences (CCS), with screening criteria set as minimum passes ≥ 5 and minPredictedAccuracy ≥ 0.9. The lima v1.7.0 software was used to separate CCS sequences of different samples based on barcode tags. Cutadapt v2.7 was then applied to remove primer sequences with a maximum allowable mismatch rate of 20%, and sequences without matched primers were discarded. Sequences were further filtered by fragment length: full-length 16S sequences ranging from 1200 to 1650 bp and full-length ITS sequences ranging from 300 to 1000 bp were retained. Chimeric sequences were subsequently removed using the UCHIME v8.1 algorithm.

USEARCH (version 10.0) was adopted to cluster sequences at the default similarity threshold of 97%, and operational taxonomic units (OTUs) with an abundance lower than 0.005% of the total sequencing reads were filtered out by default. Quality-controlled sequences were imported into QIIME2 (version 2020.6), where the DADA2 algorithm was used for (amplicon sequence variant) ASV denoising, and low-abundance ASVs accounting for less than 0.005% of total sequencing reads were discarded.

A two-step taxonomic annotation strategy was adopted. Bacterial 16S sequences were aligned against the Silva Release 138 database, while fungal ITS sequences were annotated against the UNITE Release 8.0 database. The classify-consensus-blast method was first applied for alignment-based annotation with minimum sequence similarity of 90%, minimum coverage of 90%, and consensus threshold of 51%. Sequences that failed precise alignment were further annotated via the classify-sklearn naïve Bayes classifier with a confidence cutoff of 0.7. Alpha diversity indices, including Chao1, Ace, Shannon, and Simpson, were calculated using QIIME2. The beta diversity of bacterial and fungal communities was calculated separately based on group-averaged ASV abundance data, and Bray–Curtis distance matrices were constructed for principal coordinate analysis (PCoA). Bioinformatic analyses were completed by Beijing Tsingke Biotechnology Co., Ltd. (Shanghai, China).

### 2.10. Statistical Analysis

All experiments were performed in biological triplicates (*n* = 3), with three independent tea samples prepared for each treatment group. All detections were conducted in technical triplicates to ensure experimental repeatability. One-way analysis of variance (ANOVA) was conducted using SPSS 22.0 (IBM Corp., Armonk, NY, USA), and Duncan’s multiple range test was applied for significance analysis (*p* < 0.05). Simca 14.1 was used for principal component analysis (PCA) and orthogonal partial least squares discriminant analysis (OPLS-DA) to calculate variable importance in projection (VIP). TBtools-II (v2.4) was used to draw heatmaps. Combined with Agilent Mass Profiler Professional (MPP), differential aroma components were screened under the criteria of *p* < 0.05 and VIP > 1. Correlation heatmaps were plotted via the online platform ChiPlot (https://www.chiplot.online; accessed on 20 July 2026). All correlation analyses were performed on group-averaged biological replicate data derived from 27 valid individual samples. Pearson correlation analysis was performed to explore the associations between dominant microbial taxa and tea chemical–flavor indicators. A correlation coefficient threshold of |r| ≥ 0.35 was adopted to retain moderate-to-strong biologically relevant correlations and filter out weak, trivial associations. This threshold falls within the conventional “medium effect” range (r = 0.30–0.50) defined by Cohen’s effect size guidelines and is consistent with the correlation screening standards commonly used in multi-omics microbiome–metabolite association studies [8]. The Mantel test was applied to verify the overall association between microbial community structure and metabolite indicators, and statistical significance was defined at *p* < 0.05. It should be noted that multiple-testing correction (e.g., FDR) was not applied in the present analysis; therefore, all correlation results are descriptive and exploratory rather than strictly conclusive and should be interpreted with caution. Other figures were generated using Origin Pro 2025 and Microsoft PowerPoint 2021.

## 3. Results and Discussion

### 3.1. Structural Analysis of NP

**Figure 1:**
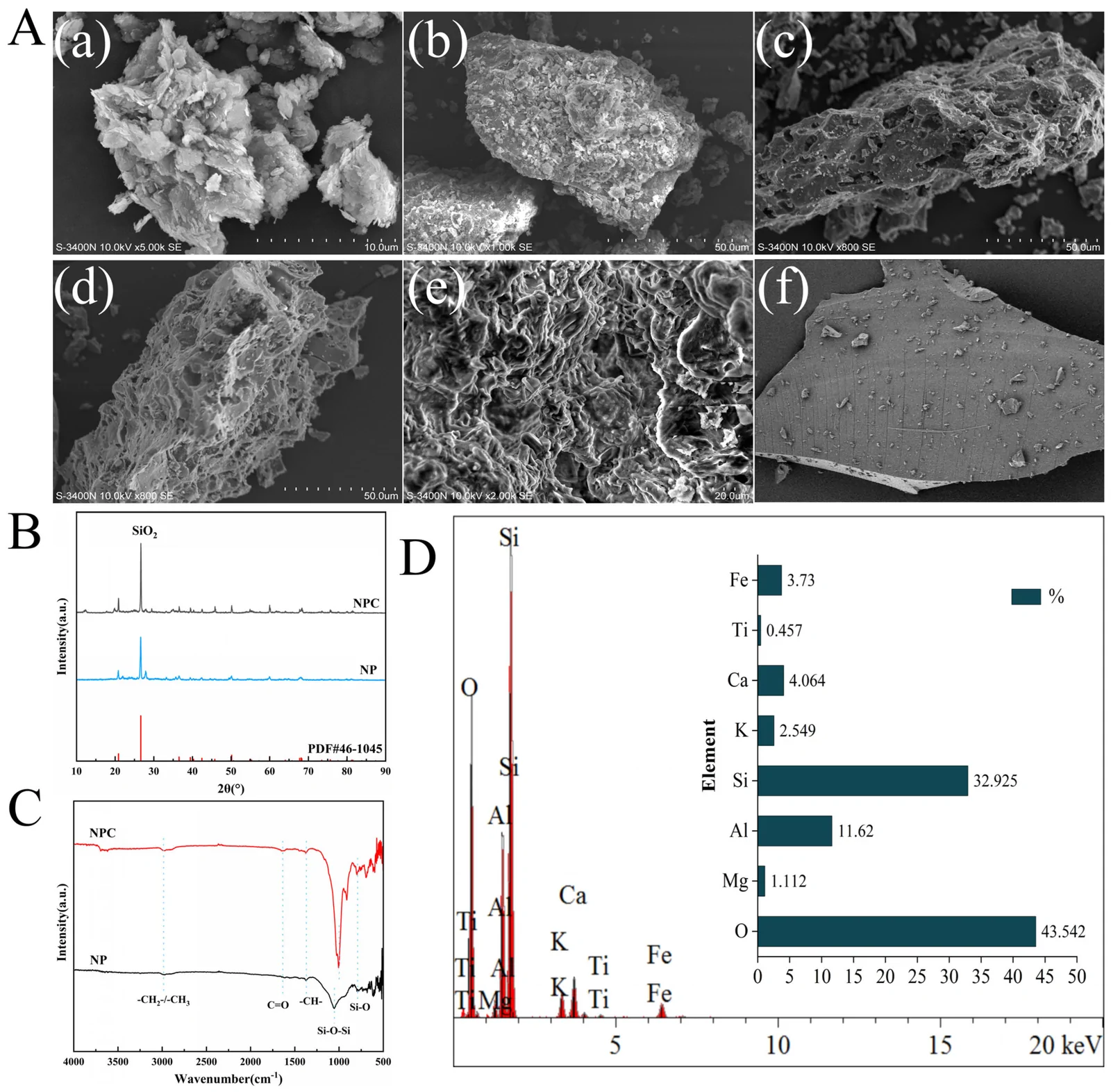
Structural characterization of Nixing Pottery and glass containers. (**A**): SEM images of NP and GC. (**a**,**b**): NP clay before firing (NPC); (**c**,**d**): NP pottery after firing (NP); (**e**): NP section (NPS); f: SEM image of GC. (**B**): XRD spectra of NPC and NP. (**C**): FTIR spectra of NPC and NP. (**D**): EDS spectrum of NP.

Figure 1A showed the SEM micrographs of NP and GC. As shown in Figure 1A (a,b), unsintered Nixing Pottery clay (NPC) consisted of loosely packed lamellar and granular aggregates with irregular open macropores. After high-temperature sintering, NP became denser with uniformly arranged fine particles, and closed or semi-closed micropores gradually formed (Figure 1A (c,d)). This phenomenon was possibly related to the sintering and shrinkage of particles caused by the dehydration of clay minerals, as well as the combustion and volatilization of organic matter at high temperatures [26]. The cross-section of NP presented a compact structure without obvious cracks or cavities (Figure 1A (e)), suggesting the presence of evenly distributed closed micropores. This distinctive microporous structure may endow NP with the property of being air-permeable but watertight. These results were consistent with the findings of Shi [27]. By comparison, GC had an amorphous, non-porous structure (Figure 1A (f)), which may hinder gas exchange, potentially leading to different storage performances between the two containers.

The XRD patterns (Figure 1B) showed that both NPC and NP mainly consist of SiO_2_, with NP displaying higher crystallinity and fewer impurities. This finding aligns with Xiao [28], who observed that the crystalline phases in fired Nixing Pottery gradually increase. The elevated crystallinity may be related to the formation of spontaneously polarized crystals, which may generate a stable surface electric field and high far-infrared emissivity [14]. The FTIR spectra (Figure 1C) indicated that residual organic matter in NP was eliminated, forming a continuous SiO_2_ framework with strengthened interparticle bonding and improved structural stability. A stable inorganic structure might prevent odor migration and chemical interactions with tea components during long-term storage. EDS elemental mapping (Figure 1D) illustrated the elemental composition and relative content of NP. It was dominated by inorganic non-metallic elements and shared similar mineral characteristics with NPC. Meanwhile, NP contained various variable-valence metal ions. This observation was consistent with the findings of Shi [29] regarding the elemental and phase composition of Nixing Pottery. These metal ions might participate in the redox reactions of tea components.

### 3.2. Effects of NP Storage on Water Quality and Full Width at Half Maximum

**Figure 2:**
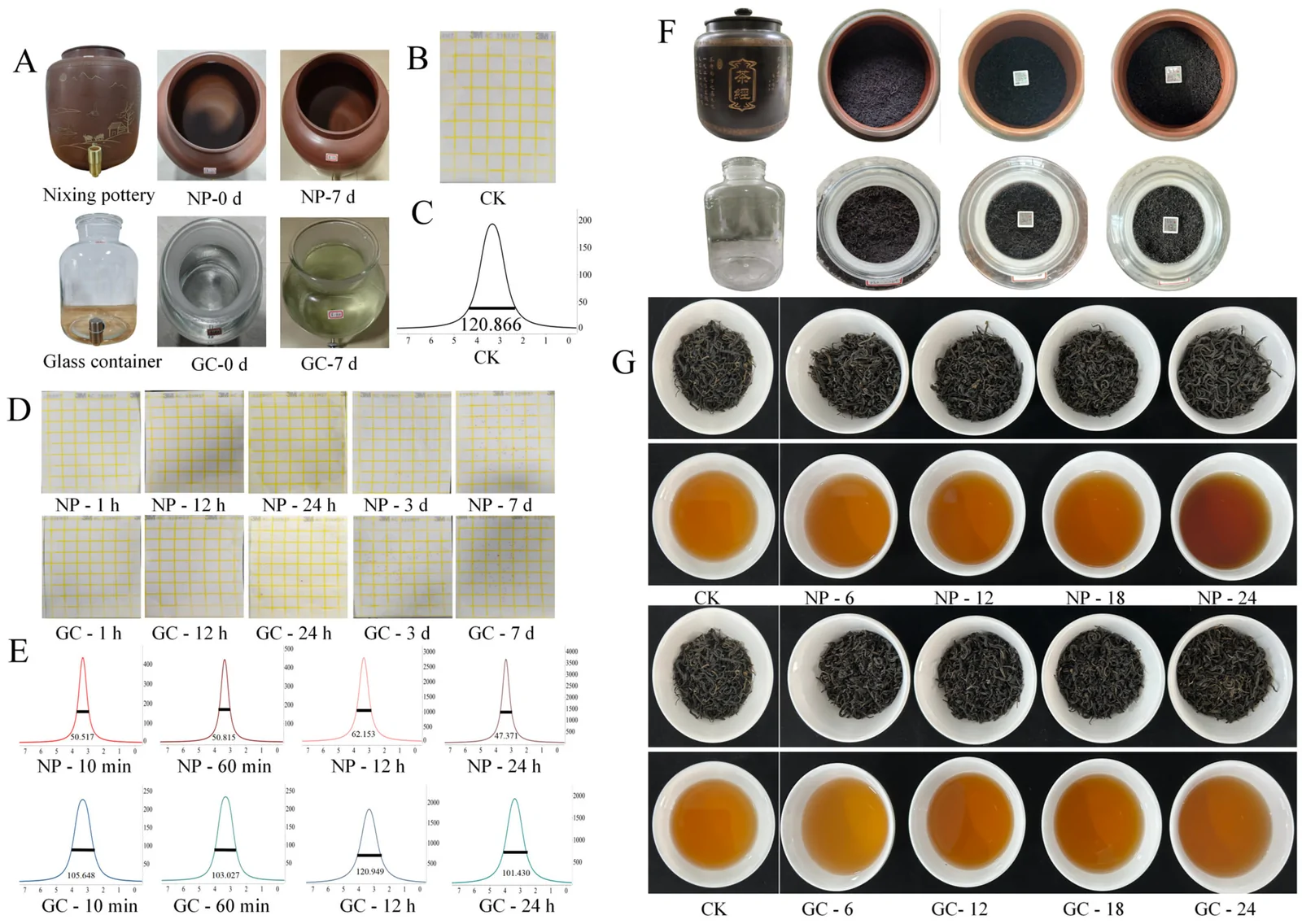
Comparison of water quality, tea samples, and tea infusion color differences under NP and GC storage. (**A**): Water storage situation (0–7 days). (**B**): Colony count of CK. (**C**): Full width at half maximum (FWHM) of CK. (**D**): Change in colony count in stored water of NP and GC. (**E**): Changes in FWHM of stored water in NP and GC. (**F**): Storage container; storage conditions of Liupao tea; temperature and humidity results. (**G**): Liupao tea samples and infusion colors during the storage period (0–24 months).

To verify the storage potential of NP, the growth of colony count and the change in full width at half maximum (FWHM) of water stored in NP and GC were examined. As shown in Figure 2A, after 7 days of storage, visible floating colonies appeared on the water surface stored in GC, and the water quality appeared pale green and turbid. In contrast, no floating colonies were observed on the water surface in NP, which maintained relatively good water quality.

The total number of bacterial colonies in water samples stored for different times was measured using colony counting test strips (Figure 2B,D). The colony count of water stored in NP remained 0 CFU/mL within 1–24 h, which was significantly different from that of GC stored for 24 h (*p* < 0.05). For GC-stored water, the colony count increased from 0 CFU/mL initially to 40 CFU/mL after 24 h. With the extension of storage time, the colony counts of water stored in NP and GC were 82 and 455 CFU/mL at 3 d and 104 and 629 CFU/mL at 7 d, respectively. These results indicated that NP may have a substantial advantage in bacteriostasis. This might be related to the physical properties of natural minerals. Rogers [30] reported that beneficial nutrients and metals in natural minerals could affect microorganisms, which was similar to our present results.

The ^17^O-NMR signal with a spin quantum number of 1/2 can be used to measure water cluster size, and a smaller FWHM indicates a smaller water cluster size [31]. The NMR results (Figure 2C,E) showed that the initial FWHM of H_2_O was 120.866 Hz, which was identified as large-molecule clustered water [32]. At the same storage time, there was a significant difference in the FWHM of water stored in NP and GC (*p* < 0.05). The FWHM of water stored in NP within 1–24 h (47.371–62.153 Hz) was significantly lower than that of water stored in GC. The results aligned with those reported by Zhang [14]. These results suggested that NP might weaken or break the hydrogen bonds between H_2_O molecules via surface electric field and spontaneous polarization. Its high far-infrared emissivity may also play a role in disrupting water clusters and forming small-molecule water [33,34]. This might be associated with the high far-infrared emissivity of pottery materials, which could play a role in disrupting water clusters, forming small-molecule water, and generating biological effects.

### 3.3. Effects of NP Storage on Color Differences of Tea and Tea Infusion

Figure 2F illustrates the appearance of NP and GC containers, the morphological state of Liupao tea after 24 months of storage, as well as the differences in internal temperature and humidity of the two containers. Liupao tea stored in NP containers presents a deep red color without obvious white foreign substances on the surface, while a pale white layer adheres to the surface of Liupao tea stored in GC containers, which is speculated to possibly be the growth of abnormal bacterial colonies.

Temperature and humidity test results indicate that the storage temperature inside NP containers is 0.2–0.6 °C lower than that of GC containers, with no significant difference between the two groups. However, the relative humidity inside NP containers ranges from 57% to 58%, whereas the humidity inside GC containers reaches 65–67%. Such discrepancy may be related to the microporous structure of mineral materials [35]. A study conducted by Gonda [36] verified that variations in temperature and humidity conditions can significantly affect tea quality, which may explain the divergent storage performances of NP and GC containers.

In addition to aroma and taste, the appearance and color of tea can also reflect tea quality to a certain extent [37]. In this study, the infusion color of Liupao tea stored in NP and GC containers for different durations was compared and analyzed. As shown in Table 1, storage using NP and GC containers exerts a significant influence on the infusion color of Liupao tea (*p* < 0.05). As storage time extends, the L* lightness value of tea infusion in the NP group shows an overall upward trend. After 24 months of storage, the NP-24 group possesses remarkably higher infusion lightness (L* value) than the GC-24 group. The b* yellowness values of both NP and GC groups fluctuate drastically with significant intergroup differences and generally shift toward yellow tones. Although the a* redness values fluctuate without notable intergroup differences, they gradually turn red overall. The GC group displays a narrower fluctuation range of a* values, while the NP-24 group has a higher redness value.

As presented in Figure 2G, the tea infusion of Liupao tea stored in NP containers gradually turns reddish-brown with prolonged storage, which may be attributed to the continuous increase in the content of soluble substances in tea during aging. The early color shift from yellow to orange-yellow may result from the combined effects of thearubigins and theaflavins, while the subsequent transition from orange-yellow to orange-red may stem from the gradual oxidative polymerization of thearubigins into theabrownins, rendering the tea infusion a reddish-brown hue [38]. Lee [39] also observed similar phenomena in his research. It should be noted that this study only adopted Liupao tea from a single manufacturer, single harvest season, and homogenized batch as the experimental material. Although the single-batch design minimized raw material variability and ensured the accuracy and reliability of container comparison results, it limits the universality of our findings. As a geographical indication of dark tea, Liupao tea varies greatly in tea cultivar, fermentation technology, and initial microbial community among different producing areas and manufacturers. Accordingly, the Nixing Pottery storage effects on tea quality aging and microbial succession observed in this work are batch- and condition-specific, which cannot be generalized to other dark tea varieties or all commercial Liupao tea products. Future studies with multi-batch and multi-origin tea samples are necessary to verify the repeatability and universality of the current conclusions.

### 3.4. Effects of NP Storage on Main Chemical Components

WE contain various beneficial components, including water-soluble sugars, amino acids, and tea polyphenols, which jointly influence the flavor of tea [40]. As shown in Table 2, the contents of WE of Liupao tea stored in both the NP group and the GC group decreased with the extension of storage time. This may be caused by chemical reactions between TP and enzymes to form insoluble substances during the storage of Liupao tea [41]. The decrease in water-soluble extracts was more significant in the GC group (*p* < 0.05). This phenomenon may be related to the low-humidity environment and bacteriostatic properties of NP [42].

FAA and TP are key indicators for evaluating tea quality [43], and their contents continuously declined with prolonged storage time. Notably, the contents of FAA and TP decreased significantly in the GC group (*p* < 0.05), while the declining trend was relatively moderate in the NP group. A stable microbial community can regulate environmental pH and prevent excessive degradation of nutrients [44]. The TPS content kept increasing with storage time, and this rising trend was particularly obvious in the NP group. Microorganisms of Firmicutes can secrete amylase and pectinase, which are capable of decomposing insoluble polysaccharides into soluble tea polysaccharides [45]. CAT represents the most important subclass of TP. It contributes greatly to bitterness and astringency and, meanwhile, affects the color, aroma, and taste of tea [46]. The CAT content of tea stored in NP decreased significantly. This may be caused by the oxidative polymerization of CAT with oxygen to form derivatives such as theaflavins. Moderate oxygen supply may drive the normal aging of tea components. Such conditions may facilitate the growth of Firmicutes and further promote substance accumulation, which was consistent with the report by Lu [47]. CAF is a key bitter-tasting flavor substance in tea [48]. The CAF content only showed slight fluctuations as storage time increased. CAF may possess high stability under different storage conditions, which was consistent with the study by Astill [49].

### 3.5. Analysis of Volatile Components

#### 3.5.1. Effects of NP on Volatile Compounds

To investigate the effects of NP on volatile compounds in tea, volatile components of Liupao tea stored for 0 (initial Liupao tea), 6, 12, 18, and 24 months were analyzed. Volatile organic compounds (VOCs) constituted the key material basis of the aromatic quality of Liupao tea, and their types and contents directly determined the flavor quality of Liupao tea [50]. As shown in Table 3, a total of 128 volatile components were identified. Among these, alcohols, aldehydes, and ketones were the predominant volatile compounds, which aligned with the findings of Ma [51].

Esters and ketones contributed fruity and woody notes to Liupao tea [52]. The ester content in Liupao tea from NP-6 was relatively high and significantly higher than that in GC-6, indicating that NP may exert an enrichment effect on esters during the early storage stage, which may be related to the adsorption characteristics of mineral materials toward ester compounds [53]. With prolonged storage time, the ester content in NP decreased sharply, while the contents of alcohols, aldehydes, and ketones increased gradually. This was attributed to environmental factors and internal biochemical reactions in tea leaves, in which most esters were gradually decomposed into other aroma components such as alcohols and aldehydes [54]. Linalyl acetate made up over 80% of the total ester content in the later stage and became the main ester component, which aligned with the findings of Wu [55]. Alcohols were dominated by cedrol, 1-octen-3-ol, and phytol. In Liupao tea stored in NP, the content of 1-octen-3-ol showed a steady upward trend, whereas an abnormal surge was observed in GC-24 (22.32%). A low content of this compound could reduce fishy and musty odors, while an excessive amount led to an aged, putrid odor in Liupao tea [56,57]. The abnormal increase of 1-octen-3-ol in GC might be caused by microorganisms and the excessive degradation of nutrients.

Aldehydes and ketones were important auxiliary components in the aroma system of Liupao tea [58]. Among them, 2,6-dimethyl-5-heptenal and 1-Cyclohexene-1-carboxaldehyde,2,6,6-trimethyl- were the core dominant components, together accounting for more than 75% of the total aldehyde content. Among ketones, 2-Butanone,4-(2,6,6-trimethyl-2-cyclohexen-1-yl)- showed the highest content in the NP-24 sample, accounting for 59.53% of the total ketones, which was significantly higher than that in GC-24. This compound was an important precursor of ionone and a key aroma component of Liupao tea [59]. The relatively stable catechins and tea polysaccharides in NP might supply sufficient precursors for the synthesis of characteristic ketones [60].

Heterocyclic compounds harmonize the overall aroma through interactions with alcohols, esters, and other VOCs [61]. Heterocyclic compounds were closely related to microbial metabolism. The balanced microbial community and stable chemical composition in NP might jointly facilitate their accumulation. These results collectively indicate that significant differences exist in the flavor components of Liupao tea stored in NP and GC.

#### 3.5.2. Multivariate Statistical Analysis of Volatile Components

To further understand the similarities and differences in flavor components between Liupao tea stored in NP and GC, principal component analysis (PCA) was performed based on volatile compound data. PCA was a multivariate statistical method that extracted a small number of significant variables through linear transformation of multiple variables. It allowed evaluation of patterns and variability among samples based on the contributions of principal component factors across different samples [62].

**Figure 3:**
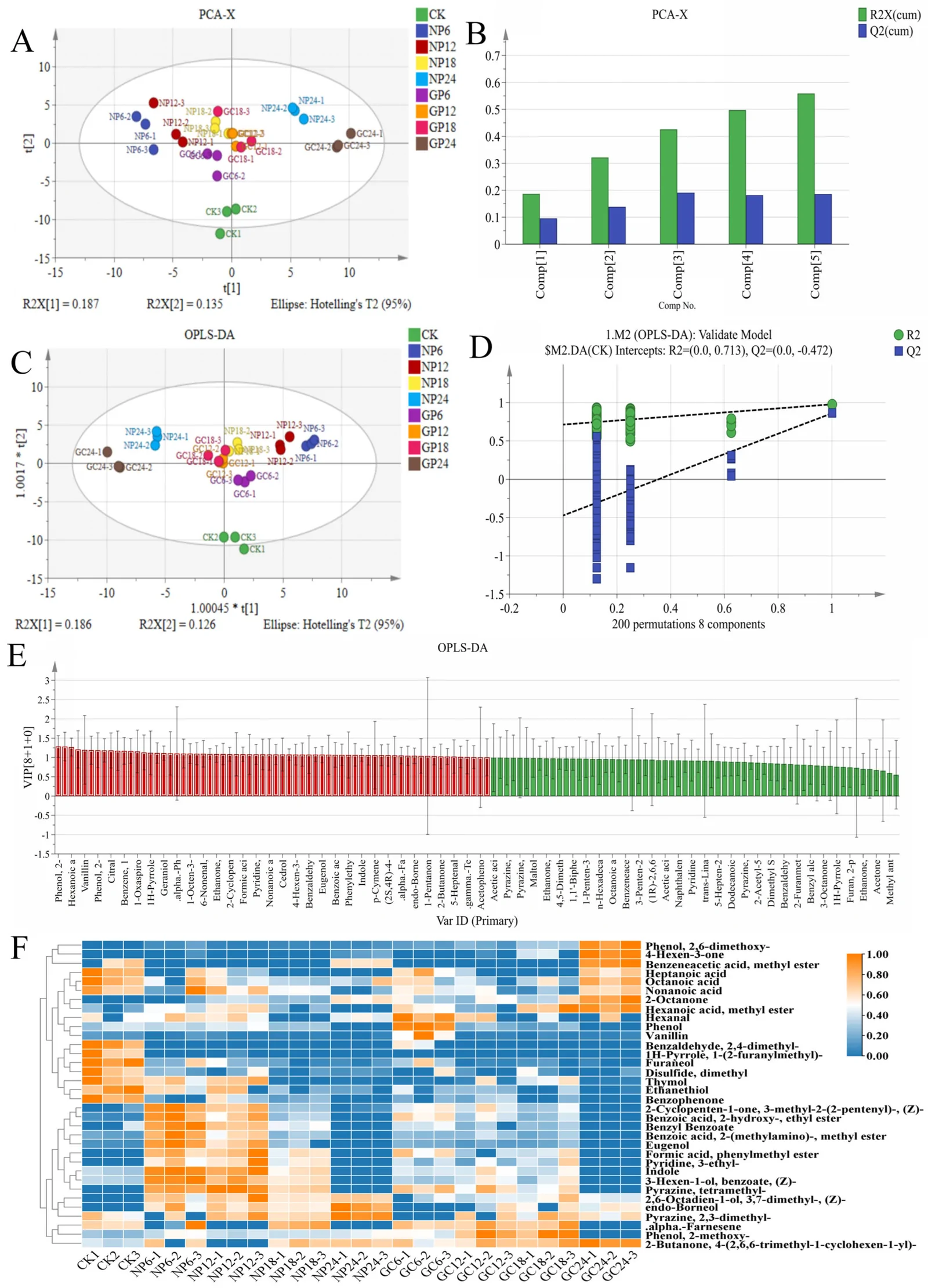
Results of multivariate statistical analysis of volatile compounds in Liupao tea. (**A**): PCA score plot. (**B**): Cumulative contribution plot. (**C**): OPLS-DA score plot. (**D**): Permutation test plot with 200 iterations. (**E**): VIP value distribution of volatile compounds. (**F**): Cluster heatmap of differential volatile compounds (*p* < 0.05, VIP > 1).

Multivariate statistical analysis of volatile components from nine samples (including eight samples and CK) showed that (Figure 3A) the contribution rates of PC1 and PC2 were 32.6%. The first two principal components did not fully capture all information, possibly because of influences from factors other than storage conditions. NP and GC samples displayed distinct clustering patterns at different storage stages. CK was relatively close to GC-6, and GC-6, GC-12, and GC-18 overlapped to some extent, suggesting a relatively slow aging process. The slow aging of GC samples might result from its unfavorable microenvironment, while NP’s stable storage conditions might accelerate the evolution of flavor substances [63].

When the principal components were further increased (Figure 3B), the cumulative contribution rate of the first five principal components reached 55.8%, which could reasonably reflect the main characteristics of the samples [64]. Accordingly, supervised OPLS-DA was further conducted. As shown in Figure 3C, the fitness indices (R^2^X = 0.700, R^2^Y = 0.945) and predictive index (Q^2^ = 0.631) of the OPLS-DA model were obtained. R^2^X and R^2^Y represent the explanatory power of the model for the X and Y matrices, respectively, and Q^2^ indicates the predictive ability of the model. Values closer to 1 indicate a more stable and reliable model, and values above 0.5 for R^2^ and Q^2^ suggest acceptable model fitting. To further verify model reliability, 200 permutation tests were performed (Figure 3D). The intercept of the Q^2^ regression line on the y-axis was less than 0, indicating that the model was valid and free from overfitting and thus suitable for discriminant analysis among groups.

In the OPLS-DA model, VIP scores for each volatile compound were used to identify flavor components that significantly contribute to Liupao tea, aiding the detection of differential markers. Generally, VIP > 1 indicates that volatile compounds make important contributions to Liupao tea quality [65]. As shown in Figure 3E, a total of 66 volatile compounds with VIP > 1 (marked in red) were identified as key differential components contributing greatly to Liupao tea, including 4-(2,6,6-trimethyl-1-cyclohexen-1-yl)-2-butanone, cis-3,7-dimethyl-2,6-octadien-1-ol, and others. Qin [66] reported that core microorganisms were positively correlated with the formation of key aroma substances in Liupao tea. Therefore, the differences in their contents might be regulated by the structural properties and microbial effects of NP.

#### 3.5.3. Analysis of Differential Volatile Components

To further clarify the effect of NP on volatile compounds in Liupao tea, the standardized content data of volatile compounds from all samples were used to screen 34 differential aroma compounds based on the criteria of *p* < 0.05 and VIP > 1. A hierarchical clustering heatmap was constructed to reveal the clustering patterns and differential characteristics of volatile compounds in Liupao tea between NP and GC groups.

The heatmap used a color gradient to intuitively reflect the relative expression levels of flavor compounds, with blue representing low expression and orange representing high expression [67]. As shown in Figure 3F, each group exhibited good within-group correlation and high clustering compactness. The CK sample formed an independent and tightly clustered branch, which was clearly separated from all treatment groups, indicating that the overall composition of volatile compounds in Liupao tea underwent fundamental changes after storage. NP-6 and NP-12 clustered into one group; GC-6, GC-12, GC-18, and NP-18 clustered into another group; and NP-24 and GC-24 each formed separate clusters. The clustering results were nearly consistent with those from PCA. The color gradient distribution of the NP heatmap revealed clear stage characteristics during storage, consistent with the aging properties of Liupao tea [68]. Within 6–12 months, the volatile compounds of Liupao tea experienced a relatively gentle change. In contrast, from 12 to 24 months, the composition and structure of volatile compounds were significantly restructured, ultimately forming a flavor system distinctly different from that in the early storage stage.

For the GC heatmap, flavor compounds changed slightly within 6–18 months, indicating a slow aging process, which led to significant differences in flavor composition compared with NP-stored Liupao tea. Furthermore, GC-24 showed a specific highly expressed cluster (intensive orange region) composed of several acids (benzeneacetic acid, heptanoic acid, and octanoic acid) and phenols (2,6-dimethoxyphenol), which was notably distinct from NP-24. This phenomenon may be related to oxidative deterioration, which could lead to disorganized and unpleasant tea aroma [69].

### 3.6. Effects of NP Storage on Microorganisms in Liupao Tea Samples

To comprehensively investigate the dynamic evolution of microorganisms during the storage of Liupao tea in NP, specific primers were used to amplify the fungal ITS gene and bacterial 16S rDNA gene regions by PCR, followed by high-throughput sequencing (PacBio). Liupao tea samples stored in NP and GC for 0 (initial tea sample), 6, 12, 18, and 24 months were tested.

The α-diversity of microbial communities was comprehensively assessed by calculating the Chao1 (community richness estimator), Simpson (Simpson diversity index), Shannon (Shannon–Wiener diversity index), and ACE (abundance-based coverage estimator) indices [70]. As shown in Table 4, the Chao1 index of Liupao tea samples stored in NP decreased over time. It was significantly higher than that of GC during the early storage stage (0–12 months), but the Chao1 index of NP-24 was notably lower than that of GC-24 (*p* < 0.05). The ACE index exhibited a similar trend to the Chao1 index, whereas the Simpson and Shannon indices first increased and then decreased, yet both were significantly lower than those of GC at 24 months. These results aligned with those reported by Gan [71], who found that bacterial abundance in Liupao tea steadily decreased during aging. Compared with bacteria, the overall fungal diversity in NP decreased over storage time, while in GC, it increased, indicating significant differences in fungal community composition between Liupao tea stored in NP and GC at different stages (*p* < 0.05).

**Figure 4:**
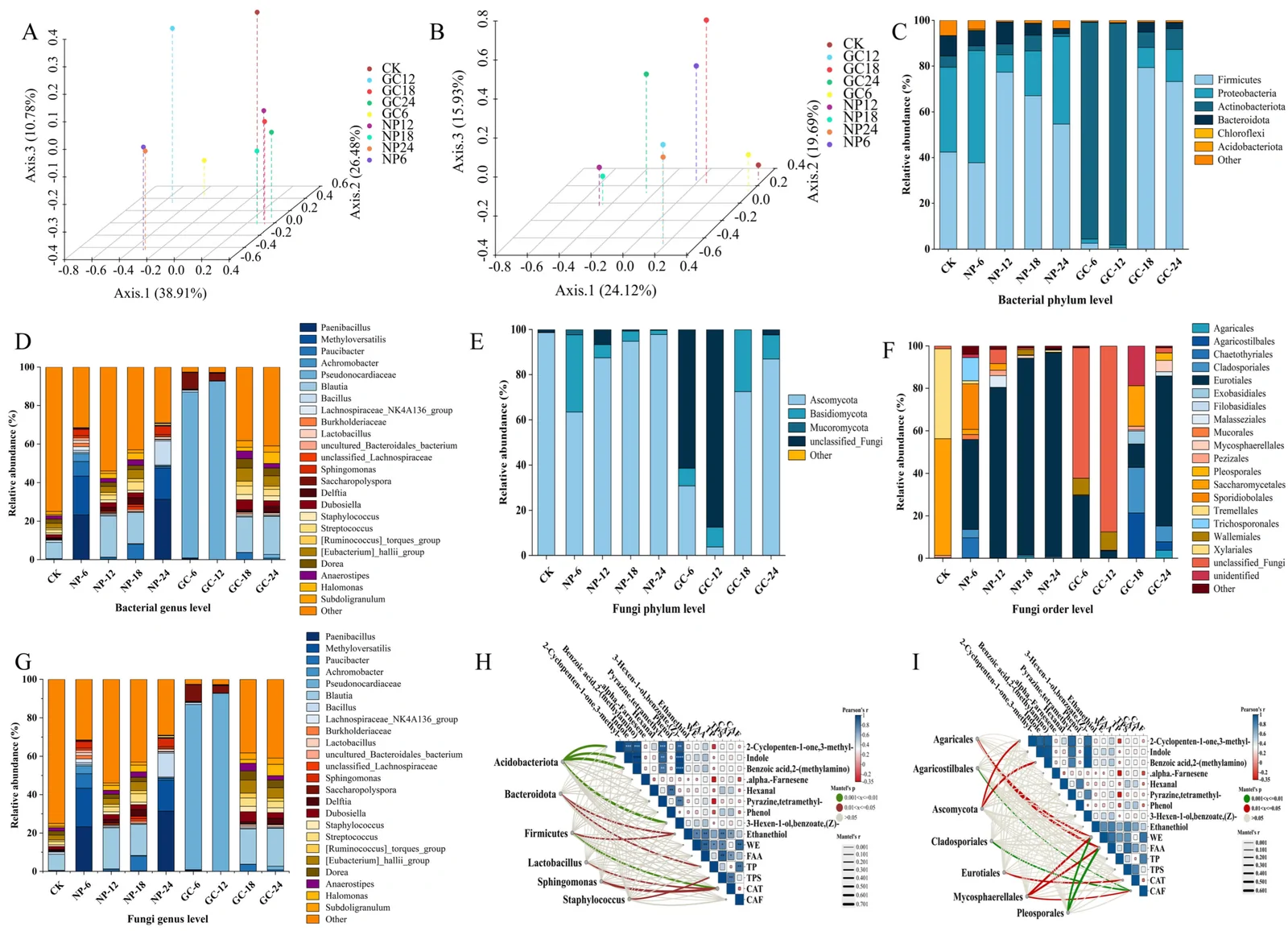
Comparative analyses of beta diversity, microbial community composition, relative abundance, and correlation patterns of bacteria and fungi in Liupao tea samples stored under NP and GC conditions. (**A**): PCoA analysis of bacterial communities. (**B**): PCoA analysis of fungal communities. (**C**): Bacterial community composition at the phylum level. (**D**): Bacterial community composition at the genus level. (**E**): Fungal community composition at the phylum level. (**F**): Fungal community composition at the order level. (**G**): Fungal community composition at the genus level. (**H**): Pearson correlation heatmap between bacterial taxa and volatile/non-volatile compounds. (**I**): Pearson correlation heatmap between fungal taxa and volatile/non-volatile compounds. (Blue squares in the upper-right heatmap represent positive Pearson correlations, and red squares indicate negative Pearson correlations; only correlations with |r| ≥ 0.35 are exhibited. In the lower-left Mantel network, line thickness corresponds to the magnitude of Mantel’s r value, and line colors denote different significance levels of Mantel’s *p*-values (green: 0.001 < *p* ≤ 0.01; red: 0.01 < *p* ≤ 0.05; gray: *p* ≥ 0.05). The statistical significance threshold for Mantel test was set at *p* < 0.05.

Three-dimensional PCoA analysis of bacterial communities based on Bray–Curtis distance (Figure 4A) showed that the first principal component explained 38.91% of the total variation, the second principal component explained 26.48%, and the third principal component explained 10.78%, with a cumulative explanatory rate of 76.17%. The microbial community structures of CK and GC-12 samples were highly similar, while the community structures of other treatment groups (GC-18, GC-24, GC-6, NP-12, NP-18, NP-24, and NP-6) changed significantly and were clearly separated from the control group. Three-dimensional PCoA analysis of fungal communities based on Bray–Curtis distance (Figure 4B) revealed that the microbial community composition of CK was significantly different from all GC and NP groups, and the CK group was distinctly separated from other samples. The community structures under the two storage modes (GC and NP) partially overlapped yet presented a certain separation trend, which suggested that storage mode exerted an influence on microbial communities.

### 3.7. Analysis of Microbial Community Structure and Composition

The composition and relative abundance of dominant bacterial and fungal were further examined to clarify the differences in microbial community structure between NP and GC during storage.

As shown in Figure 4C, at the phylum level, Firmicutes (42.42%), Proteobacteria (37.12%), and Bacteroidota (8.91%) were the dominant phyla in CK, accounting for more than 90% of total bacteria, which aligned with the findings of Weng [72]. During the 0–24 month storage period, Liupao tea stored in NP maintained a relatively balanced distribution of these three phyla. In NP-6, Firmicutes made up 37.72%, and Proteobacteria comprised 49.09%, while at the genus level (Figure 4D), *Paenibacillus* (23.23%) and *Methyloversatilis* (20.16%) were the most dominant genera. Stable Firmicutes communities in NP were accompanied by better-retained tea polysaccharides and reduced losses of amino acids and tea polyphenols [73], which was consistent with the results of TPS and TP.

In contrast, GC-6 and GC-12 samples were dominated by Firmicutes and Actinobacteriota, among which Actinobacteriota accounted for more than 90% of the total bacteria. The excessively high abundance of this phylum was observed; members of this phylum have been reported to produce off-flavor substances such as earthy-smelling geosmin [74] and strongly inhibit the growth of other bacterial phyla simultaneously. Correspondingly, the relative abundance of *Pseudonocardiaceae* increased substantially, reaching 86.10%, and this taxon belonged taxonomically to Actinobacteriota. Some members within this family can secrete abundant carbohydrate-degrading enzymes, including cellulase, which might play vital roles in the metabolism of tea substrates [75]. The markedly upregulated abundance of Actinobacteriota in the GC group was paralleled by the degradation of endogenous tea components and the appearance of earthy and musty off notes, and the remarkable elevation in the contents of volatile compounds such as 1-octen-3-ol in the GC group might be correlated with this observation. Furthermore, *Pseudonocardiaceae* can colonize fresh tea leaves as endophytic actinobacteria and generally maintain a low relative abundance under normal conditions [76]. No dramatic upregulation or downregulation of Actinobacteriota and *Pseudonocardiaceae* abundances was observed in NP-12, NP-18, and NP-24 samples, which only presented slight variations.

For fungi (Figure 4E), Ascomycota and Basidiomycota were the dominant phyla, which were consistent with the results reported by Nong [77]. *Eurotium cristatum*, known as the “golden flower” fungus in dark tea, belonged to the family Ascomycotaceae [78]. As an aerobic microorganism, *Eurotium cristatum* might require a higher oxygen level [79]. At all storage stages, the relative abundance of Ascomycota in Liupao tea stored in NP was significantly higher than that in GC, and the same trend was observed for Eurotiales at the order level (Figure 4F). Although the abundance of Ascomycota in GC-24 recovered to 86.91%, it was still lower than that in NP-24 (97.76%). In addition, GC accumulated a large number of other fungal phyla within 0–12 months, which led to a remarkable reduction in the relative abundance of Ascomycota. At the order level, the relative abundances of Cladosporiales and other microorganisms were significantly upregulated in GC-24, which might indicate potential risks [80]. At the genus level (Figure 4G), the relative abundance of *Aspergillus* [81] in NP-24 reached 10.06%, which was significantly higher than that in GC-24. The relative abundance of this genus was highly correlated with the variation in the abundance of Eurotiales. It has been reported to secrete various extracellular enzymes and possess capabilities for polyphenol oxidation and accumulation of flavor substances [82]. Meanwhile, more microbial genera were detected, or their abundances were upregulated in GC-18 and GC-24 samples, including *Alternaria*, *Cladosporium*, and *Exobasidium*. Such a fungal community structure with altered abundances might cause oxidative deterioration of tea leaves. Sour taste and phenolic off odors were identified in the heatmap analysis of volatile components, which was consistent with the findings reported by Cui [83].

Liupao tea stored in NP maintained relatively stable communities of Firmicutes and Proteobacteria within 24 months of storage, and the relative abundances of other microorganisms were significantly suppressed. This might be related to the microporous structure of NP. NP created microaerobic conditions and an ambient humidity of approximately 57%, both of which favored tea fermentation. This finding was consistent with the study conducted by Xu [84].

The results of this study indicated that there were obvious differences in the changes of community structure between Liupao tea stored under NP and GC conditions. NP might be more conducive to the preservation, storage, and aging of Liupao tea and had the potential to alleviate nutrient loss, flavor deterioration, and microbial contamination caused by inappropriate warehousing conditions, providing a scientific basis for manufacturers to select storage containers for finished tea products.

### 3.8. Correlation Analysis Between Dominant Microbial Populations and Compounds

To investigate the regulatory role of microorganisms during Liupao tea storage, correlation analysis was performed between 13 dominant microbial taxa (6 bacterial taxa and 7 fungal taxa) and non-volatile components, as well as differential volatile metabolites (screening criteria: *p* < 0.05, VIP > 1) under NP and GC storage treatments. A total of 34 differential volatile compounds were annotated, and 9 core characteristic volatile substances were finally screened for subsequent association analysis.

As shown in Figure 4H, among bacterial taxa, Acidobacteriota was significantly positively correlated with (Z)-3-methyl-2-(2-pentenyl)cyclopent-2-en-1-one, indole, methyl 2-(methylamino)benzoate, and (Z)-3-hexen-1-yl benzoate. Bacteroidota and Firmicutes were significantly positively correlated with ethanethiol (*p* < 0.05). This result aligned with the functional characteristics of Acidobacteriota in degrading polysaccharides in lignocellulose-rich substrates, and the increase in its relative abundance might promote the accumulation of soluble sugars in tea infusion [85]. Firmicutes, *Lactobacillus*, and *Sphingomonas* were significantly positively correlated with CAT content. Bacteroidota was significantly positively correlated with TP content. *Staphylococcus* was significantly positively correlated with TPS content. These microorganisms play roles in transforming macromolecular substances and promoting the conversion of polyphenols [9,54,86]. The results indicated that stable enrichment of Firmicutes and Bacteroidota in NP corresponded to a distinct correlative network associated with TPS, TP, and CAT. This provides a plausible observational explanation for the relatively moderate TP loss and sustained TPS accumulation, although mechanistic causality remains to be verified.

As shown in Figure 4I, fungi showed significant influences on WE, FAA, and CAF. Agaricales, Mycosphaerellales, and Pleosporales were all significantly positively correlated with WE and FAA contents. Agaricostilbales, Cladosporiales, and Mycosphaerellales were significantly positively correlated with CAF content. Ascomycota was significantly positively correlated with (Z)-3-methyl-2-(2-pentenyl)cyclopent-2-en-1-one and indole. Eurotiales were positively correlated with CAT and maintained high abundance in NP. The above results indicated that obvious differences existed in tea storage performance between NP and GC groups.

## 4. Conclusions

The NMR results revealed that water stored in NP exhibited a significantly lower FWHM value and suppressed bacterial growth compared with the GC group within 24 h of storage. During the 24-month storage trial, NP slowed the degradation of non-volatile substances (WE, FAA, and TP) in Liupao tea and shaped distinct volatile compound profiles relative to GC. Microbiological analysis demonstrated that NP maintained a relatively stable microbial community dominated by Firmicutes, Bacteroidota, and Ascomycota. Correlation analysis indicated that these core microbial taxa were closely associated with the transformation of tea chemical components.

The above results indicated that significant differences existed in tea storage performance between the NP group and the GC group. This study provided a theoretical basis for the fresh keeping of high-quality tea, the optimization of post-fermentation technology, and the popularization of Nixing Pottery containers and also offered new insights into the development of novel natural mineral-based packaging materials. However, multiple potential working mechanisms were involved when Nixing Pottery regulated the aging process of tea, including slow aeration through micropores, humidity adjustment via water absorption of the mineral matrix, and biological effects generated by the surface spontaneous polarization electric field. Meanwhile, the present study did not conduct comprehensive sensory evaluation, which imposes limitations on the interpretation of some of our research findings. Future targeted in-depth mechanistic investigations combined with thorough sensory assessments should be performed to systematically elucidate the potential core regulatory processes of Nixing Pottery during the long-term storage of tea.

## Figures and Tables

**Table 1:** Variation of L*, a*, and b* values in Liupao tea and infusion with different storage containers.

Sample	L*	a*	b*
CK	9.27 ± 0.12 ^ab^	1.13 ± 0.12 ^a^	−0.87 ± 0.17 ^a^
NP-6	9.43 ± 0.12 ^abc^	1.20 ± 0.36 ^a^	−0.30 ± 0.29 ^abc^
NP-12	9.73 ± 0.19 ^cd^	0.90 ± 0.24 ^a^	0.23 ± 0.21 ^abc^
NP-18	9.53 ± 0.09 ^bc^	1.13 ± 0.26 ^a^	−0.03 ± 0.24 ^abc^
NP-24	9.97 ± 0.12 ^d^	1.47 ± 0.21 ^a^	0.80 ± 0.08 ^c^
GC-6	9.03 ± 0.29 ^a^	1.30 ± 0.57 ^a^	−0.57 ± 0.98 ^ab^
GC-12	9.40 ± 0.22 ^abc^	0.70 ± 0.22 ^a^	0.53 ± 0.42 ^bc^
GC-18	9.73 ± 0.25 ^cd^	1.00 ± 0.65 ^a^	−0.07 ± 0.88 ^abc^
GC-24	9.17 ± 0.12 ^ab^	1.13 ± 0.12 ^a^	0.13 ± 0.17 ^abc^

Note: Different lowercase letters within the same column indicate significant differences at *p* < 0.05 determined by one-way analysis of variance (ANOVA). Abbreviations: NP, Nixing Pottery; GC, glass container; CK, control group (initial tea sample); 6, 12, 18, 24 represent storage time in months; L*, brightness; a*, redness; b*, yellowness.

**Table 2:** Changes in main chemical components of Liupao tea under different storage containers.

Sample	Content (mg/g)					
	WE	FAA	TP	TPS	CAT	CAF
CK	43.73 ± 0.91 ^a^	3.71 ± 0.14 ^a^	19.89 ± 0.29 ^a^	4.10 ± 0.15 ^a^	14.46 ± 0.07 ^a^	3.52 ± 0.07 ^a^
NP-6	42.11 ± 1.23 ^b^	3.39 ± 0.39 ^ab^	17.52 ± 0.34 ^bc^	3.66 ± 0.08 ^b^	12.28 ± 0.12 ^c^	3.46 ± 0.02 ^a^
NP-12	41.66 ± 0.74 ^b^	3.12 ± 0.27 ^bc^	17.27 ± 0.91 ^bc^	3.11 ± 0.09 ^c^	10.95 ± 0.46 ^d^	3.49 ± 0.02 ^a^
NP-18	40.91 ± 0.42 ^bc^	2.92 ± 0.04 ^c^	17.34 ± 1.20 ^bc^	2.93 ± 0.13 ^c^	9.48 ± 0.23 ^e^	3.54 ± 0.03 ^a^
NP-24	40.12 ± 0.16 ^cd^	2.27 ± 0.07 ^d^	18.68 ± 1.02 ^ab^	2.89 ± 0.05 ^c^	6.72 ± 0.13 ^f^	3.57 ± 0.03 ^a^
GC-6	39.06 ± 0.31 ^de^	3.10 ± 0.04 ^bc^	16.01 ± 0.24 ^cde^	3.73 ± 0.21 ^b^	13.62 ± 0.13 ^b^	3.15 ± 0.02 ^b^
GC-12	37.85 ± 0.42 ^e^	2.77 ± 0.03 ^c^	15.68 ± 0.21 ^de^	3.14 ± 0.08 ^c^	12.07 ± 0.35 ^c^	3.03 ± 0.08 ^bc^
GC-18	35.24 ± 0.48 ^f^	2.28 ± 0.19 ^d^	14.94 ± 0.29 ^e^	2.63 ± 0.06 ^d^	9.97 ± 0.15 ^e^	2.99 ± 0.12 ^c^
GC-24	33.72 ± 0.58 ^g^	1.55 ± 0.20 ^e^	16.60 ± 0.39 ^cd^	2.47 ± 0.08 ^d^	9.85 ± 0.12 ^e^	3.00 ± 0.07 ^c^

Note: Different lowercase letters within the same column indicate significant differences at *p* < 0.05, as determined by one-way analysis of variance (ANOVA). Abbreviations: NP, Nixing Pottery; GC, glass container; CK, control group (initial tea sample); 6, 12, 18, 24 represent storage time in months; WE, water extract; FAA, free amino acids; TP, tea polyphenols; TPS, tea polysaccharide; CAT, catechin; CAF, caffeine.

**Table 3:** Changes in volatile organic compounds of Liupao tea stored under NP and GC conditions at different storage times (relative percentage content: %). All values listed in Table 3 denote relative peak area percentages without absolute concentration units.

NO.	Volatile Compound	CAS #	Retention Time (Rt min)	Retention Index (RI)	CK	NP-6	NP-12	NP-18	NP-24	GC-6	GC-12	GC-18	GC-24
**Alcohols**													
1	Cedrol	77-53-2	33.36	2117	3.38 ± 0.52	3.73 ± 0.44	3.63 ± 0.37	3.46 ± 0.52	3.22 ± 1.37	4.11 ± 0.73	2.26 ± 0.35	2.66 ± 0.85	3.29 ± 0.38
2	1-Octen-3-ol	3391-86-4	18.81	1454	0.59 ± 1.02	1.27 ± 1.2	1.47 ± 1.63	2.13 ± 0.29	2.88 ± 0.41	0.67 ± 0.61	0.67 ± 0.74	1.41 ± 1.22	22.32 ± 0.93
3	Phytol	150-86-7	41.87	2616	2.85 ± 0.94	1.66 ± 0.75	1.99 ± 0.63	1.55 ± 0.3	0.78 ± 0.89	2.02 ± 0.63	1.29 ± 0.55	1.15 ± 0.37	0.91 ± 0.17
4	Benzyl alcohol	100-51-6	28.33	1863	11.22 ± 3.53	-	-	-	1.08 ± 0.22	9.27 ± 8.11	6.47 ± 11.08	5.26 ± 9.12	0.24 ± 0.03
5	cis-3,7-dimethyl-2,6-octadien-1-ol	106-25-2	27.06	1803	-	0.36 ± 0.11	0.46 ± 0.14	0.33 ± 0.01	0.39 ± 0.02	0.14 ± 0.13	0.21 ± 0.03	0.32 ± 0.11	0.23 ± 0.02
6	Phenylethyl alcohol	60-12-8	29.31	1910	1.36 ± 1.27	2.21 ± 2.46	4.41 ± 2.56	4.63 ± 0.83	2.2 ± 0.42	3.91 ± 0.27	1.6 ± 1.53	2.77 ± 0.28	1.2 ± 0.13
7	2-Furanmethanol, 5-ethenyltetrahydro-.alpha.,.alpha.,5-trimethyl-, cis-	5989-33-3	18.52	1443	1.03 ± 0.89	-	1.27 ± 2.2	2.95 ± 2.57	2.22 ± 0.21	0.81 ± 1.4	-	2.17 ± 1.88	0.62 ± 1.07
8	Geraniol	106-24-1	28.01	1848	0.52 ± 0.25	-	-	0.53 ± 0.46	0.19 ± 0.33	0.71 ± 0.02	0.89 ± 0.14	0.5 ± 0.17	-
9	endo-Borneol	507-70-0	24.56	1690	-	0.8 ± 0.33	0.83 ± 0.14	0.66 ± 0.02	1.23 ± 0.21	-	0.37 ± 0.38	0.44 ± 0.39	0.61 ± 0.12
10	1-Penten-3-ol	616-25-1	10.73	1176	1.68 ± 1.47	0.11 ± 0.19	-	1.26 ± 2.19	1.83 ± 3.03	-	2.95 ± 0.56	1.16 ± 2.01	-
11	Terpinen-4-ol	562-74-3	22.52	1602	-	-	0.18 ± 0.31	0.18 ± 0.31	1.32 ± 0.18	-	-	-	-
12	trans-Linalool oxide (furanoid)	34995-77-2	19.18	1469	-	-	-	2.52 ± 4.36	1.27 ± 0.03	-	0.93 ± 1.61	-	0.49 ± 0.86
**Aldehydes**													
13	Benzaldehyde, 2,4-dimethyl-	15764-16-6	27.10	1805	0.66 ± 0.11	-	-	-	-	0.23 ± 0.2	-	-	-
14	Benzaldehyde	100-52-7	22.94	1620	0.94 ± 0.2	1.77 ± 3.06	0.6 ± 0.1	2.09 ± 2.89	0.16 ± 0.27	0.99 ± 1.19	1.05 ± 1.07	0.39 ± 0.11	0.14 ± 0.25
15	Hexanal	66-25-1	7.50	1076	0.95 ± 0.84	1.75 ± 0.53	1.58 ± 0.45	-	1.14 ± 0.22	3.34 ± 0.84	1.42 ± 1.24	0.37 ± 0.65	0.71 ± 1.23
16	2,6-dimethyl-5-heptenal	106-72-9	14.84	1310	3.42 ± 0.37	1.99 ± 1.83	1.39 ± 2.41	4.32 ± 0.54	9.8 ± 1.24	2.57 ± 0.52	2.99 ± 2.7	4.95 ± 1.52	3.04 ± 5.27
17	1H-Pyrrole-2-carboxaldehyde, 1-methyl-	1192-58-1	29.35	1912	0.24 ± 0.34	0.04 ± 0.02	-	0.02 ± 0	0.02 ± 0	0.11 ± 0.11	0.01 ± 0	0.02 ± 0	0 ± 0.01
18	Benzaldehyde, 2-hydroxy-	90-02-8	24.09	1670	0.03 ± 0.06	0.12 ± 0.02	0.13 ± 0.03	0.08 ± 0.02	-	0.05 ± 0.08	0.04 ± 0.04	0.05 ± 0.08	-
19	Vanillin	121-33-5	40.93	2557	0.03 ± 0	0.02 ± 0.01	0.02 ± 0.01	0.01 ± 0	0.01 ± 0	0.14 ± 0.06	0.01 ± 0	0.01 ± 0.01	0.02 ± 0
20	2-Octenal, (E)-	2548-87-0	18.05	1426	-	0.83 ± 0.72	0.81 ± 0.73	0.98 ± 0.14	0.1 ± 0.17	0.6 ± 1.05	0.89 ± 0.11	0.61 ± 0.69	-
21	1,3-Cyclohexadiene-1-carboxaldehyde, 2,6,6-trimethyl-	116-26-7	23.44	1642	1.76 ± 1.53	2.72 ± 0.36	3.48 ± 0.37	1.37 ± 1.2	2.65 ± 2.3	1.44 ± 1.3	2.12 ± 0.45	2.22 ± 0.64	-
22	Citral	5392-40-5	25.46	1730	-	0.13 ± 0.02	-	0.04 ± 0.07	-	-	0.02 ± 0.03	-	-
23	1-Cyclohexene-1-carboxaldehyde, 2,6,6-trimethyl-	432-25-7	22.85	1617	-	-	-	2.5 ± 4.32	5.25 ± 4.55	-	1.21 ± 2.1	1.81 ± 3.14	2.09 ± 3.61
24	6-Nonenal, (Z)-	2277-19-2	20.80	1532	-	-	-	-	-	-	-	-	1.37 ± 0.15
**Ketones**													
25	2-Cyclopenten-1-one, 3-methyl-2-(2-pentenyl)-, (Z)-	488-10-8	29.96	1942	0.03 ± 0.04	0.46 ± 0.05	0.35 ± 0.04	0.13 ± 0	0.02 ± 0.02	0.23 ± 0.04	0.22 ± 0.07	0.16 ± 0.06	-
26	(+)-2-Bornanone	464-49-3	20.25	1510	-	0.43 ± 0.37	0.29 ± 0.51	0.45 ± 0.39	-	0.67 ± 0.09	0.2 ± 0.35	0.18 ± 0.32	-
27	3-Buten-2-one, 4-(2,2,6-trimethyl-7-oxabicyclo[4.1.0]hept-1-yl)-	23267-57-4	30.92	1990	1.27 ± 0.3	1.52 ± 0.05	1.75 ± 0.37	1.31 ± 0.18	1.73 ± 0.43	1.38 ± 0.19	1.31 ± 0.39	1.42 ± 0.36	1.57 ± 0.12
28	2-Octanone	111-13-7	14.03	1282	-	0.03 ± 0.05	0.17 ± 0.01	0.11 ± 0.02	0.28 ± 0.03	0.17 ± 0.07	0.19 ± 0.08	0.2 ± 0.1	0.46 ± 0.05
29	4-(2,6,6-trimethyl-1-cyclohexen-1-yl)-2-butanone	14901-07-6	29.76	1932	5.25 ± 0.31	5.24 ± 4.72	8.45 ± 1.95	6.54 ± 0.41	15.62 ± 2.24	6.56 ± 1.61	6.96 ± 1.97	0.42 ± 2.52	7.63 ± 0.84
30	2-Heptanone	110-43-0	10.84	1179	0.54 ± 0.48	0.19 ± 0.32	0.94 ± 0.14	0.58 ± 0.51	1.03 ± 0.92	0.9 ± 0.14	-	0.46 ± 0.4	0.54 ± 0.93
31	Acetophenone, 4’-hydroxy-	99-93-4	28.64	1878	-	0.09 ± 0.1	-	-	0.03 ± 0	0.01 ± 0.01	0.01 ± 0.01	0 ± 0.01	0.02 ± 0.02
32	Acetone	67-64-1	10.72	1175	0 ± 0.01	0.1 ± 0.14	0.1 ± 0.08	0.04 ± 0.05	0.03 ± 0.04	0.06 ± 0.1	0.07 ± 0.06	-	0.01 ± 0.01
33	5-Hepten-2-one, 6-methyl-	110-93-0	15.57	1335	3.75 ± 3.29	1.98 ± 3.43	4 ± 3.51	5.62 ± 0.86	3.88 ± 0.16	1.54 ± 2.67	2.61 ± 2.27	3 ± 2.69	7.04 ± 0.42
34	1-Penten-3-one	1629-58-9	9.05	1124	0.48 ± 0.55	0.06 ± 0.11	1.16 ± 0.92	0.19 ± 0.17	0.02 ± 0.02	1 ± 0.67	0.51 ± 0.89	0.34 ± 0.36	0.1 ± 0.02
35	Furaneol	3658-77-3	31.78	2034	0.16 ± 0.01	0.06 ± 0.04	0.05 ± 0.03	0.01 ± 0.01	0.01 ± 0.01	0.07 ± 0.04	0.01 ± 0	0.01 ± 0.01	0 ± 0.01
36	2-Butanone	78-93-3	4.39	/	0.07 ± 0.11	0.09 ± 0.08	0.2 ± 0.19	0.21 ± 0.04	0.51 ± 0.11	0.23 ± 0.05	0.04 ± 0.06	0.13 ± 0.12	0.25 ± 0.27
37	2-Butanone, 4-(2,6,6-trimethyl-1-cyclohexen-1-yl)-	17283-81-7	27.64	1830	0.3 ± 0.04	0.4 ± 0.35	-	0.59 ± 0.1	0.61 ± 0.04	0.48 ± 0.16	0.62 ± 0.14	0.68 ± 0.12	0.9 ± 0.07
38	Ethanone, 1-(2-hydroxy-5-methylphenyl)-	1450-72-2	34.73	2191	0.06 ± 0.01	-	-	-	0.03 ± 0.05	0.03 ± 0.04	0.02 ± 0.03	-	-
39	Benzophenone	119-61-9	39.60	2474	0.03 ± 0	-	0.01 ± 0.01	0.01 ± 0	-	-	0.01 ± 0	-	-
40	3-Penten-2-one, 4-methyl-	141-79-7	9.15	1127	1.92 ± 1.69	-	1.42 ± 1.26	0.35 ± 0.61	1.31 ± 1.16	0.67 ± 1.15	0.5 ± 0.87	2.19 ± 0.61	0.45 ± 0.78
41	3-Octanone	106-68-3	13.27	1257	-	0.94 ± 0.07	0.81 ± 0.08	0.98 ± 0.23	0.98 ± 0.02	-	0.83 ± 0.14	0.47 ± 0.09	1.03 ± 1.72
42	2-Butanone, 4-(2,6,6-trimethyl-2-cyclohexen-1-yl)-	31499-72-6	27.29	1814	-	-	-	-	0.05 ± 0.09	-	-	0.16 ± 0.15	0.82 ± 0.05
43	2-Cyclohexen-1-one	930-68-7	20.83	1533	0.01 ± 0.01	-	-	-	-	0 ± 0.01	0.01 ± 0.01	-	0.01 ± 0.01
44	4-Hexen-3-one	2497-21-4	15.32	1327	-	0.02 ± 0.03	-	-	0.08 ± 0.03	-	0.02 ± 0.03	0.17 ± 0.15	0.7 ± 0.04
45	2-Decanone	693-54-9	19.81	1492	-	-	-	-	-	-	-	-	1.31 ± 0.41
**Esters**													
46	Formic acid, phenylmethyl ester	104-57-4	24.38	1682	-	0.28 ± 0.05	0.31 ± 0.07	0.21 ± 0.04	-	0.23 ± 0.05	0.08 ± 0.07	0.17 ± 0.05	-
47	Benzoic acid, 2-(methylamino)-, methyl ester	85-91-6	32.43	2068	0.1 ± 0.02	0.7 ± 0.12	0.45 ± 0.1	0.19 ± 0.04	-	0.16 ± 0.01	0.21 ± 0.06	0.16 ± 0.06	-
48	9,12,15-Octadecatrienoic acid, methyl ester, (Z,Z,Z)-	301-00-8	41.02	2562	-	-	-	-	-	0.01 ± 0.02	-	0.07 ± 0.01	0.06 ± 0.05
49	Hexanoic acid, methyl ester	106-70-7	11.05	1186	0.05 ± 0.05	0.15 ± 0.03	0.22 ± 0.02	0.05 ± 0.05	-	0.2 ± 0.03	0.05 ± 0.04	0.29 ± 0.07	0.33 ± 0.03
50	Benzyl benzoate	120-51-4	42.01	2625	0.01 ± 0.01	0.04 ± 0	0.03 ± 0	0.02 ± 0	-	0.02 ± 0	0.01 ± 0.01	0.01 ± 0	-
51	Methyl anthranilate	134-20-3	35.49	2233	1.88 ± 0.35	3.22 ± 5.57	2.08 ± 3.6	2.71 ± 0.25	0.01 ± 0.02	2.03 ± 0.27	2 ± 0.3	1.95 ± 0.68	-
52	Benzoic acid, 2-hydroxy-, ethyl ester	118-61-6	27.11	1805	0.02 ± 0.01	0.33 ± 0.05	0.25 ± 0.07	0.11 ± 0.02	-	0.22 ± 0.03	0.14 ± 0.04	0.1 ± 0.04	-
53	3-Hexen-1-ol, benzoate, (Z)-	25152-85-6	33.47	2123	1.06 ± 0.11	6.81 ± 0.62	4.48 ± 1.21	2.62 ± 0.25	0.01 ± 0	0.96 ± 0.16	1.82 ± 0.54	1.58 ± 0.95	-
54	9,12-Octadecadienoic acid (Z,Z)-, methyl ester	112-63-0	39.94	2495	-	0.02 ± 0.02	0.03 ± 0.03	-	0.01 ± 0.02	0.02 ± 0.02	-	0.04 ± 0.03	0.05 ± 0.02
55	Methyl tetradecanoate	124-10-7	31.32	2010	0.02 ± 0	0.01 ± 0.01	0.01 ± 0.01	-	0.01 ± 0.01	0.02 ± 0.01	-	0.01 ± 0.01	-
56	Linalyl acetate	115-95-7	21.27	1551	-	-	-	0.52 ± 0.91	9.91 ± 0.85	4.58 ± 6.09	0.29 ± 0.51	-	5.3 ± 0.41
57	2-Furanmethanol, tetrahydro-, acetate	637-64-9	14.05	1283	0.09 ± 0.15	0.02 ± 0.01	-	0 ± 0.01	0.02 ± 0.02	0 ± 0.01	0.01 ± 0.01	0.04 ± 0.03	0.01 ± 0.01
58	Methyl salicylate	119-36-8	26.32	1769	1.93 ± 0.34	12.88 ± 1.84	6.24 ± 5.7	4.88 ± 0.41	0.17 ± 0.15	-	4.2 ± 4.11	4.91 ± 2.6	0.28 ± 0.02
59	Acetic acid, methyl ester	79-20-9	3.83	/	0.13 ± 0.23	0.4 ± 0.08	0.35 ± 0.27	-	-	-	-	0.17 ± 0.28	-
60	Butanoic acid, 3-hexenyl ester, (Z)-	16491-36-4	30.11	1950	0.15 ± 0.03	0.11 ± 0.19	-	0.1 ± 0.08	-	-	-	0.07 ± 0.12	-
61	Benzoic acid, 2-methylpropyl ester	120-50-3	31.11	1999	0 ± 0.01	0.12 ± 0.03	0.07 ± 0.02	0.02 ± 0.04	-	-	-	0.02 ± 0.03	-
62	Benzeneacetic acid, methyl ester	101-41-7	26.04	1756	0.23 ± 0.2	-	-	-	0.31 ± 0.01	-	-	-	0.52 ± 0.04
**Aromatics**													
63	p-Cymene	99-87-6	16.54	1370	0.52 ± 0.45	0.37 ± 0.33	0.28 ± 0.49	1.86 ± 2.57	-	0.59 ± 0.08	0.16 ± 0.15	2.29 ± 3.42	0 ± 0.01
64	.alpha.-Farnesene	502-61-4	25.78	1745	3.85 ± 0.33	3.81 ± 6.13	-	6.32 ± 0.98	-	4.45 ± 0.87	5.47 ± 1.98	5.83 ± 2.4	-
65	1,1’-Biphenyl, 4-methyl-	644-08-6	31.85	2038	0.01 ± 0.01	-	-	-	-	0.01 ± 0	-	0.01 ± 0	-
**Olefins**													
66	.alpha.-Phellandrene	99-83-2	10.45	1167	0.24 ± 0.22	0.15 ± 0.13	0.22 ± 0.08	0.23 ± 0.02	0.47 ± 0.2	0.21 ± 0.02	0.15 ± 0.13	0.53 ± 0.52	0.15 ± 0.02
67	.gamma.-Terpinene	99-85-4	13.53	1266	2.08 ± 0.19	1.54 ± 0.18	1.83 ± 0.33	1.28 ± 0.12	2.21 ± 0.05	1.11 ± 0.18	1.3 ± 0.14	1.5 ± 0.4	1.27 ± 0.81
68	3-Carene	13466-78-9	11.80	1209	1.14 ± 1.87	0.02 ± 0.03	1.73 ± 2.99	2.15 ± 0.3	1.29 ± 0.04	0.96 ± 0.99	1.99 ± 0.19	0.94 ± 0.97	0.55 ± 0.48
69	1,3-Cyclohexadiene, 1-methyl-4-(1-methylethyl)-	99-86-5	11.25	1192	-	0.66 ± 0.97	1.26 ± 1.17	0.64 ± 0.55	1.63 ± 0.05	0.27 ± 0.46	1.08 ± 1.19	1.12 ± 0.3	0.51 ± 0.09
70	Naphthalene, 1,2,3,5,6,7,8,8a-octahydro-1,8a-dimethyl-7-(1-methylethenyl)-, [1R-(1.alpha.,7.beta.,8a.alpha.)]-	4630-07-3	24.24	1676	1.03 ± 0.3	0.08 ± 0.07	0.58 ± 1	0.04 ± 0.07	-	-	-	-	-
71	(1R)-2,6,6-Trimethylbicyclo[3.1.1]hept-2-ene	7785-70-8	18.38	1438	6.7 ± 4.93	-	-	-	-	-	-	-	-
72	D-Limonene	5989-27-5	11.79	1209	5.42 ± 8.16	3.09 ± 5.36	2.67 ± 4.62	-	-	-	4.43 ± 4.18	3.63 ± 6.29	10.71 ± 9.34
**Phenols**													
73	Phenol, 2,6-dimethoxy-	91-10-1	32.86	2090	-	0.03 ± 0.01	0.01 ± 0	0.02 ± 0	0.02 ± 0.01	0.04 ± 0.01	0.05 ± 0.02	0.08 ± 0	0.27 ± 0.02
74	p-Cresol	106-44-5	32.64	2079	0.06 ± 0.01	0.22 ± 0.02	0.23 ± 0.04	0.18 ± 0.02	0.08 ± 0	0.08 ± 0	0.07 ± 0.01	0.12 ± 0.03	0.05 ± 0.05
75	Eugenol	97-53-0	34.23	2164	0.06 ± 0.01	0.85 ± 0.09	0.62 ± 0.16	0.15 ± 0.14	-	0.15 ± 0.01	0.11 ± 0.03	0.14 ± 0.06	-
76	Phenol, 2-methoxy-	90-05-1	28.18	1856	0.07 ± 0.01	0.13 ± 0.03	0.1 ± 0.01	0.11 ± 0	0.13 ± 0.02	0.13 ± 0.02	0.25 ± 0.05	0.27 ± 0.05	-
77	Phenol	108-95-2	29.09	1899	1.12 ± 0.34	1.07 ± 0.13	1.49 ± 0.22	0.79 ± 0.07	-	6.08 ± 0.59	0.63 ± 0.2	0.47 ± 0.23	0.01 ± 0.02
78	Maltol	118-71-8	30.35	1962	0.24 ± 0.04	0.27 ± 0.16	0.27 ± 0.07	0.2 ± 0.07	0.15 ± 0.1	0.22 ± 0.12	0.04 ± 0.05	0.11 ± 0.05	0.11 ± 0.04
79	Thymol	89-83-8	24.75	1698	0.46 ± 0.11	0.33 ± 0.09	0.41 ± 0.05	-	0.01 ± 0	0.01 ± 0.01	0.12 ± 0.18	0.26 ± 0.06	0.01 ± 0
80	Phenol, 2-methyl-	95-48-7	27.38	1818	5.88 ± 5.8	-	-	5.16 ± 8.94	-	4.2 ± 7.27	13.85 ± 7.39	4.8 ± 8.31	-
81	Benzene, 1,2-dimethoxy-4-(1-propenyl)-	93-16-3	34.51	2179	-	0.02 ± 0	0.02 ± 0.02	-	-	-	-	-	-
82	Phenol, 4-ethyl-	123-07-9	14.83	1309	0 ± 0.01	-	0.02 ± 0.03	0.02 ± 0.02	0.06 ± 0.02	-	0.02 ± 0.04	0.02 ± 0.02	0.19 ± 0.01
83	Phenol, 4-ethyl-2-methoxy-	2785-89-9	30.91	1989	-	-	-	-	-	-	0.64 ± 1.08	0.01 ± 0.01	0.05 ± 0
84	Phenol, 2-methyl-5-(1-methylethyl)-	499-75-2	17.64	1410	-	-	-	-	0.35 ± 0.05	-	-	-	-
**Ethers**													
85	Benzene, 1-methoxy-4-methyl-	104-93-8	12.90	1245	0.42 ± 0.06	0.17 ± 0.16	0.35 ± 0.1	-	-	0.12 ± 0.21	0.08 ± 0.14	0.23 ± 0.2	0.16 ± 0.27
86	Benzene, 1,3-dimethoxy-	151-10-0	25.75	1743	-	-	-	0.08 ± 0.07	0.2 ± 0.03	-	0.15 ± 0.02	-	-
**Acids**													
87	Heptanoic acid	111-14-8	30.27	1958	0.2 ± 0.03	0.05 ± 0.09	0.08 ± 0.04	-	-	0.15 ± 0.04	0.03 ± 0.04	-	0.15 ± 0.02
88	Octanoic acid	124-07-2	32.39	2066	0.25 ± 0.04	0.16 ± 0.09	0.16 ± 0.03	0.13 ± 0.01	0.16 ± 0.05	0.17 ± 0.05	0.11 ± 0.05	0.14 ± 0.04	0.24 ± 0.02
89	n-Decanoic acid	334-48-5	36.32	2280	0.01 ± 0.02	-	0.04 ± 0.01	0.05 ± 0	0.03 ± 0.01	0.03 ± 0.01	0.04 ± 0.01	0.03 ± 0.01	0.04 ± 0.01
90	n-Hexadecanoic acid	57-10-3	46.37	2921	0.23 ± 0.05	0.1 ± 0.09	0.29 ± 0.04	0.11 ± 0.02	0.32 ± 0.29	0.13 ± 0.05	0.13 ± 0.13	0.1 ± 0.04	0.24 ± 0.01
91	Benzeneacetic acid	103-82-2	41.49	2592	0.31 ± 0.1	0.28 ± 0.18	0.3 ± 0.14	0.15 ± 0.05	0.19 ± 0.13	0.23 ± 0.25	0.06 ± 0.06	0.12 ± 0.11	0.25 ± 0.05
92	Nonanoic acid	112-05-0	34.40	2173	0.42 ± 0.05	0.31 ± 0.2	0.4 ± 0.06	0.32 ± 0.01	0.17 ± 0.04	0.34 ± 0.04	0.27 ± 0.09	0.19 ± 0.05	0.39 ± 0.02
93	Acetic acid	64-19-7	18.70	1450	-	-	0.14 ± 0.25	0.21 ± 0.22	0.87 ± 0.77	0.47 ± 0.13	-	0.25 ± 0.06	0.21 ± 0.03
94	Pentanoic acid	109-52-4	26.45	1775	0.29 ± 0.06	0.29 ± 0.49	0.23 ± 0.21	0.21 ± 0.36	0.16 ± 0.07	0.12 ± 0.1	0.07 ± 0.06	0.07 ± 0.06	1.04 ± 0.18
95	Geranic acid	459-80-3	37.44	2345	0.01 ± 0.01	0.01 ± 0.01	0.02 ± 0.01	0.01 ± 0	0.01 ± 0.01	0.01 ± 0.01	0.01 ± 0.01	-	0.01 ± 0.01
96	Dodecanoic acid	143-07-7	39.91	2492	-	-	0.01 ± 0.01	-	0.02 ± 0.01	-	-	0.01 ± 0.01	0.01 ± 0.01
**Heterocyclic compounds**													
97	Pyrazine	290-37-9	12.21	1223	0.02 ± 0	0.03 ± 0	0.04 ± 0.01	0.16 ± 0.2	0.12 ± 0	0.04 ± 0.01	0.03 ± 0.02	0.03 ± 0	0.02 ± 0.02
98	Indole	120-72-9	38.01	2378	0.79 ± 0.14	4.6 ± 0.32	3.99 ± 0.88	1.96 ± 0.37	0.02 ± 0.01	1.09 ± 0.09	1.58 ± 0.57	1.14 ± 0.38	0.01 ± 0.01
99	Pyrazine, ethyl-	13925-00-3	15.43	1331	0.48 ± 0.42	0.53 ± 0.05	0.68 ± 0.05	0.53 ± 0.03	0.72 ± 0.04	0.84 ± 0.33	0.36 ± 0.31	0.6 ± 0.08	0.47 ± 0.04
100	Caffeine	58-08-2	49.95	3147	3.86 ± 1.47	3.8 ± 2.37	3.66 ± 1.95	3.17 ± 1.12	3.01 ± 2.1	3.94 ± 1.39	2.52 ± 1.78	3.26 ± 1.03	3.46 ± 0.61
101	Pyridine, 3-ethyl-	536-78-7	16.77	1378	-	0.11 ± 0.03	0.14 ± 0.04	0.1 ± 0.02	-	0.06 ± 0.02	0.05 ± 0.03	0.08 ± 0.04	-
102	Ethanone, 1-(1H-pyrrol-2-yl)-	1072-83-9	30.48	1968	2.51 ± 2.2	5.14 ± 1.04	4.03 ± 3.51	3.31 ± 2.86	4.76 ± 2.98	4.28 ± 3.97	2.63 ± 2.34	4.56 ± 0.9	5.22 ± 1.9
103	Furan, 2-pentyl-	3777-69-3	12.42	1229	4.77 ± 4.13	4.9 ± 4.32	6.45 ± 5.63	2.97 ± 5.14	1.98 ± 3.42	7.11 ± 1.38	3.74 ± 3.24	2.69 ± 2.44	1.71 ± 2.96
104	1-Pentanone, 1-(2-furanyl)-	3194-17-0	23.48	1643	-	0.01 ± 0.02	0.01 ± 0.01	0.02 ± 0.01	0.03 ± 0.05	0.01 ± 0.01	0.26 ± 0.39	0.01 ± 0.01	-
105	Dimethyl sulfoxide	67-68-5	21.86	1575	0.72 ± 0.2	0.51 ± 0.16	0.58 ± 0.3	0.48 ± 0.2	0.27 ± 0.29	0.71 ± 0.13	0.47 ± 0.19	0.46 ± 0.13	0.18 ± 0.31
106	1-Oxaspiro[4.5]dec-6-ene, 2,6,10,10-tetramethyl-	36431-72-8	20.87	1535	1.11 ± 1.08	2.91 ± 0.5	3.56 ± 0.66	0.4 ± 0.35	2.21 ± 0.18	1.62 ± 0.31	1.73 ± 0.47	1.93 ± 0.64	2.03 ± 0.27
107	Pyrazine, tetramethyl-	1124-11-4	19.36	1475	-	2.03 ± 0.15	2.54 ± 0.17	1.37 ± 0.13	-	1.53 ± 0.26	1.33 ± 0.52	0.96 ± 0.32	-
108	2-Acetyl-5-methylfuran	1193-79-9	13.17	1254	0.02 ± 0.03	0.03 ± 0.02	0.07 ± 0.02	0.06 ± 0.01	0.08 ± 0.01	0.04 ± 0.03	0.07 ± 0.05	0.05 ± 0.01	0.04 ± 0.01
109	Pyrazine, 2-(n-propyl)-	18138-03-9	17.80	1416	0.01 ± 0.01	0.02 ± 0	0.02 ± 0	0.01 ± 0	0.02 ± 0	0.02 ± 0	0.01 ± 0.01	0.02 ± 0	0.01 ± 0.01
110	Benzothiazole	95-16-9	30.12	1950	0.23 ± 0.4	0.15 ± 0.02	0.12 ± 0.11	0.09 ± 0.08	0.02 ± 0.04	0.11 ± 0.1	0.15 ± 0.03	0.06 ± 0.11	-
111	4,5-Dimethyl-2-isobutyloxazole	26131-91-9	15.97	1350	0.03 ± 0.01	0.02 ± 0	0.01 ± 0.01	0.01 ± 0	-	0.02 ± 0	0.01 ± 0.01	0.02 ± 0	-
112	Furan, 2-ethyl-	3208-16-0	3.97	/	2.24 ± 0.45	1.16 ± 0.36	1.44 ± 0.15	1.32 ± 0.08	2.12 ± 0.22	1.62 ± 0.47	1.58 ± 0.55	0.98 ± 0.85	0.79 ± 0.12
113	5-Thiazoleethanol, 4-methyl-	137-00-8	36.80	2307	-	-	-	-	-	-	-	-	-
114	5H-5-Methyl-6,7-dihydrocyclopentapyrazine	23747-48-0	22.94	1620	0 ± 0.01	0.02 ± 0	0.02 ± 0.01	0.01 ± 0.01	0.02 ± 0	0.02 ± 0	0.01 ± 0	0.01 ± 0.01	-
115	Pyrazine, 2-ethyl-6-methyl-	13925-03-6	16.95	1384	-	0.2 ± 0.17	0.27 ± 0.23	1.1 ± 0.68	0.35 ± 0.02	0.23 ± 0.2	0.09 ± 0.15	0.56 ± 0.97	0.29 ± 0.1
116	2-n-Butyl furan	4466-24-4	10.05	1155	-	0.06 ± 0.1	0.12 ± 0.11	0.21 ± 0.03	0.13 ± 0.11	0.47 ± 0.64	0.25 ± 0.04	0.55 ± 0.66	0.17 ± 0.01
117	Ethanethiol	75-08-1	1.96	/	1.27 ± 0.3	0.68 ± 0.21	0.68 ± 0.59	0.18 ± 0.16	0.11 ± 0.1	0.27 ± 0.24	0.27 ± 0.03	0.13 ± 0.22	0.02 ± 0.03
118	Pyrazine, methyl-	109-08-0	13.40	1262	0.54 ± 0.17	0.62 ± 0.05	0.73 ± 0.09	0.63 ± 0.04	0.88 ± 0.07	0.77 ± 0.05	0.68 ± 0.15	0.72 ± 0.07	0.65 ± 0.07
119	Ethanone, 1-(2-furanyl)-	1192-62-7	18.77	1453	-	1.82 ± 0.18	-	1.03 ± 0.89	-	1.19 ± 1.03	-	1.08 ± 0.65	0.5 ± 0.87
120	7-Oxabicyclo[2.2.1]heptane, 1-methyl-4-(1-methylethyl)-	470-67-7	22.53	1603	-	0.18 ± 0.32	0.38 ± 0.33	-	-	-	0.38 ± 0.33	0.68 ± 0.29	0.69 ± 0.62
121	Ethanone, 1-(1-methyl-1H-pyrrol-2-yl)-	932-16-1	23.67	1652	-	0.09 ± 0.01	0.04 ± 0.07	0.06 ± 0.05	-	-	0.03 ± 0.05	0.01 ± 0.01	-
122	Pyridine	110-86-1	10.81	1178	-	0.05 ± 0.08	0.06 ± 0.11	0.13 ± 0.01	0.03 ± 0.05	-	0.04 ± 0.07	0.06 ± 0.11	-
123	Pyrazine, 2,3-dimethyl-	5910-89-4	15.79	1343	0.08 ± 0.02	0.03 ± 0.05	0.1 ± 0.02	0.08 ± 0	0.12 ± 0.01	-	0.06 ± 0.05	0.08 ± 0.03	0.07 ± 0.01
124	1H-Pyrrole, 1-(2-furanylmethyl)-	1438-94-4	27.53	1825	0.06 ± 0.02	-	-	-	-	-	-	-	-
125	Disulfide, dimethyl	624-92-0	7.12	1064	0.04 ± 0.01	0.01 ± 0	0.01 ± 0.01	-	0.01 ± 0	0.01 ± 0.02	-	0.01 ± 0.01	0 ± 0.01
126	2-Ethyl-3-methoxypyrazine	25680-58-4	18.16	1430	-	-	0.01 ± 0.01	0 ± 0.01	0.01 ± 0	-	0 ± 0.01	-	-
127	Pyrazine, 3,5-diethyl-2-methyl-	18138-05-1	19.84	1494	-	0.15 ± 0.25	-	-	-	0.15 ± 0.25	-	0.11 ± 0.2	0.27 ± 0.04
128	(2S,4R)-4-Methyl-2-(2-methylprop-1-en-1-yl)tetrahydro-2H-pyran	3033-23-6	26.42	1774	-	-	0.04 ± 0.07	0.03 ± 0.05	-	-	-	-	0.14 ± 0.01

Note: Abbreviations: NP, Nixing Pottery; GC, glass container; CK, control group (initial tea sample); 6, 12, 18, 24 represent storage time (months); CAS, Chemical Abstracts Service Registry Number; RT, retention time; RI, retention index; The symbol “-” indicates the compound was not detected (below the instrumental detection limit). Values denote relative peak-area percentages obtained by peak-area normalization, rather than absolute concentrations. Owing to differences in ionization efficiency among volatile compounds, numerical comparisons between different compounds are not valid. Only relative changes of the identical compound across groups and time points should be interpreted. Relatively large standard deviations observed for some volatile components were attributed to heterogeneous distribution of trace volatile substances among biological replicates. All raw instrumental data and statistical results have been checked and verified.

**Table 4:** Effects of NP and GC storage on microbial α-diversity indices of Liupao tea samples at different storage stages.

Microbial Category	Sample	Chao 1	Simpson	Shannon	ACE
Bacteria	CK	563.11 ± 30.18 ^a^	0.99 ± 0.01 ^a^	7.31 ± 0.94 ^a^	553.74 ± 27.23 ^a^
	NP-6	365.50 ± 34.52 ^b^	0.90 ± 0.05 ^bc^	5.25 ± 0.43 ^cd^	362.90 ± 35.13 ^b^
	NP-12	316.00 ± 30.21 ^bc^	0.98 ± 0.01 ^ab^	6.41 ± 0.23 ^ab^	311.12 ± 29.86 ^bc^
	NP-18	304.91 ± 30.21 ^bc^	0.97 ± 0.02 ^ab^	6.33 ± 0.38 ^ab^	300.75 ± 40.88 ^bc^
	NP-24	191.00 ± 24.49 ^d^	0.86 ± 0.03 ^c^	4.41 ± 0.34 ^d^	190.58 ± 24.25 ^d^
	GC-6	136.17 ± 14.65 ^d^	0.50 ± 0.02 ^d^	1.73 ± 0.13 ^e^	139.42 ± 15.18 ^d^
	GC-12	278.09 ± 27.44 ^c^	0.39 ± 0.02 ^e^	1.28 ± 0.22 ^e^	283.71 ± 27.90 ^c^
	GC-18	352.55 ± 25.33 ^b^	0.97 ± 0.02 ^ab^	5.93 ± 0.40 ^bc^	341.80 ± 24.93 ^bc^
	GC-24	272.87 ± 32.35 ^c^	0.97 ± 0.02 ^ab^	6.09 ± 0.61 ^bc^	279.64 ± 33.72 ^c^
Fungus	CK	42.50 ± 1.63 ^bcd^	0.57 ± 0.02 ^bc^	1.46 ± 0.06 ^c^	76.88 ± 2.96 ^a^
	NP-6	57.00 ± 9.80 ^a^	0.88 ± 0.15 ^a^	3.70 ± 0.64 ^a^	51.07 ± 8.78 ^bcd^
	NP-12	47.43 ± 2.08 ^abc^	0.49 ± 0.02 ^c^	1.71 ± 0.07 ^c^	50.28 ± 2.20 ^bcd^
	NP-18	46.00 ± 6.53 ^abc^	0.17 ± 0.02 ^d^	0.74 ± 0.11 ^c^	48.34 ± 6.86 ^bcd^
	NP-24	40.00 ± 2.45 ^cd^	0.30 ± 0.02 ^d^	1.25 ± 0.08 ^cd^	40.39 ± 2.47 ^d^
	GC-6	32.50 ± 4.08 ^d^	0.58 ± 0.07 ^bc^	1.85 ± 0.23 ^c^	40.89 ± 5.14 ^cd^
	GC-12	56.20 ± 4.90 ^a^	0.23 ± 0.02 ^d^	0.74 ± 0.06 ^c^	53.99 ± 4.71 ^bc^
	GC-18	54.00 ± 8.16 ^ab^	0.87 ± 0.13 ^a^	3.24 ± 0.49 ^a^	61.52 ± 9.30 ^b^
	GC-24	57.17 ± 3.97 ^a^	0.67 ± 0.05 ^b^	2.49 ± 0.17 ^b^	60.72 ± 4.22 ^b^

Note: Different lowercase letters within the same column indicate significant differences at *p* < 0.05, determined by one-way ANOVA. Abbreviations: NP, Nixing Pottery; GC, glass container; CK, control check (initial tea sample); 6, 12, 18, 24 represent storage time (months); Chao 1, community richness estimator; Simpson, Simpson diversity index; Shannon, Shannon–Wiener diversity index; ACE, abundance-based coverage estimator.

## Data Availability

The data that support the findings of this study are available from the corresponding author upon reasonable request. The datasets generated and analysed during the current study are available in the SRA (NCBI) repository, [https://www.ncbi.nlm.nih.gov/bioproject/PRJNA1504928; accessed on 25 July 2026].
